# Fibrotic Changes and Endothelial-to-Mesenchymal Transition Promoted by VEGFR2 Antagonism Alter the Therapeutic Effects of VEGFA Pathway Blockage in a Mouse Model of Choroidal Neovascularization

**DOI:** 10.3390/cells9092057

**Published:** 2020-09-09

**Authors:** Franco Aparecido Rossato, Yu Su, Ashley Mackey, Yin Shan Eric Ng

**Affiliations:** 1 Department of Ophthalmology, Schepens Eye Research Institute of Mass Eye and Ear, and Harvard Medical School, 20 Staniford Street, Boston, MA 02114, USA; rossatofap@gmail.com (F.A.R.); sy_daisy1206@163.com (Y.S.); ashley.mackey@biogen.com (A.M.); 2 Ophthalmic Center, Renmin Hospital of Wuhan University, Wuhan 430000, China

**Keywords:** endothelial-to-mesenchymal transition, choroidal neovascularization, age-related macular degeneration, VEGFA, VEGFA resistance

## Abstract

Many patients with wet age-related macular degeneration do not respond well to anti- vascular endothelial growth factor A (VEGFA) therapy for choroidal neovascularization (CNV), and the efficacy of anti-VEGFA decreases over time. We investigated the hypothesis that fibrotic changes, in particular via endothelial-to-mesenchymal transition (EndoMT), play a role in CNV and alter the therapeutic effects of VEGFA pathway blockage. Induction of EndoMT of primary human retinal endothelial cells led to a significantly reduced response to VEGFA at the level of gene expression, cellular proliferation, migration, and tube formation. Suppression of EndoMT restored cell responsiveness to VEGFA. In a mouse model of spontaneous CNV, fibrotic changes and EndoMT persisted as the CNV lesions became more established over time. VEGFA receptor-2 (VEGFR2) antagonism further induced fibrosis and EndoMT in the CNV. The combination of VEGFR2 antagonism and fibrosis/EndoMT inhibition was more effective than either individual treatment in reducing CNV. Our data indicate that fibrosis and EndoMT are involved in the progression of CNV, are exacerbated by VEGFR2 inhibition, and could provide an explanation for the reduced efficacy of anti-VEGFA treatment over time.

## 1. Introduction

Age-related macular degeneration (AMD) is characterized by degeneration of the retina and retinal pigment epithelium (RPE), particularly in the macula, which severely impacts central vision [1]. The wet form of AMD, accounting for about 80% of the severe vision loss in patients with AMD, is characterized by choroidal neovascularization (CNV) in the sub-retinal space. The new vessels are highly permeable, causing retinal and sub-retinal edema and contributing to neuroretinal degeneration. Anti-vascular endothelial growth factor A (VEGFA) therapies are currently the standard treatment for CNV [2]. This approach mainly targets vascular permeability in the CNV, with some minor inhibition of neovascularization [3]. In clinical trials a large proportion of the patients with wet AMD fail to respond well to the anti-VEGFA therapy (i.e., gaining at least 15 letters in visual acuity), and a small percentage did not respond at all [4,5,6]. Furthermore, clinical data suggest a significant reduction in therapeutic efficacy of anti-VEGFA therapies after three to four years of treatment [7,8]. The mechanism(s) for non-response, poor response, and reduced efficacy over time are all unknown [9]. Because a fibrotic scar remains in the subretinal space after anti-VEGFA treatment [10], and it appears that anti-VEGFA therapy may promote fibrotic changes in the CNV [11,12], we explored the hypothesis that fibrosis and specifically endothelial-to-mesenchymal transition (EndoMT) are involved in CNV development, and that these processes reduce the efficacy of VEGFA pathway blockage in treatment of CNV.

EndoMT is a cell transdifferentiation process in which endothelial cells acquire a mesenchymal phenotype by altering gene expression. Endothelial-specific markers such as VEGFA receptor-2 (VEGFR2), CD31 and vascular endothelial cadherin (VE-cadherin) are down-regulated, while mesenchymal markers such as SNAIL-1 (SNAI1), ZEB2, vimentin, α-smooth muscle actin (α-SMA), and type I and type III collagens (COL I and COL III) are up-regulated in the endothelial cells. During normal cardiac development, EndoMT is involved in the generation of cells that give rise to the atrioventricular valves, formation of the endocardial cushion, and generation of cardiac fibroblasts and smooth muscle cells. EndoMT has recently been shown to contribute to fibrotic changes in a variety of pathologies, including atherosclerosis, cardiac fibrosis, cavernous cerebral malformations, kidney fibrosis, and pulmonary fibrosis (reviewed in [13,14]). More recently, EndoMT of the tumor vessels has been shown to participate in the development of resistance to anti-VEGFA therapy in glioblastoma, via alterations in platelet-derived growth factor (PDGF) signaling [15].

Because constant levels of VEGFA signaling maintain the endothelial phenotype and suppress EndoMT in various experimental models [13,16,17,18], we hypothesized that anti-VEGFA or other treatments to block VEGFA signaling could promote EndoMT in CNV, a fibrotic change that would be enhanced by the pro-inflammatory environment of AMD and would render the resulting CNV lesion less dependent on VEGFA for survival [19,20]. By this mechanism, EndoMT and the resulting enhanced fibrotic changes of CNV could explain reductions in responsiveness to anti-VEGFA therapies. We therefore investigated how EndoMT affects the biology of endothelial cells in response to VEGFA in vitro and identified a chemical inhibitor of this process. We then used an established mouse model of spontaneous CNV [19,21] to examine the role of EndoMT and fibrosis in CNV development, the ability of anti-VEGFR2 treatment to promote EndoMT and fibrotic changes, and the efficacy of anti-VEGFR2 therapy when used in combination with a chemical inhibitor of EndoMT. Our data support a role for EndoMT and fibrosis in CNV and in reducing the efficacy of anti-VEGFR2 treatment, and suggest that simultaneously targeting EndoMT could improve efficacy of VEGFR2 antagonism in treatment of CNV.

## 2. Materials and Methods

### 2.1. Cell Culture

Human microvascular retinal endothelial cells (hREC) (ACBRI 181—Cell Systems, Kirkland, WA, USA) were grown in complete medium (EGM-2, Lonza, Alpharetta, GA, USA) in a tissue culture incubator with 5% CO_2_ at 37 °C. Before seeding the hREC the plates were coated with 0.2% gelatin (G1890, Sigma-Aldrich, St. Louis, MO, USA) for 30 min, and the culture medium was changed every other day. The cells were utilized between passages 5 and 9 for all experiments. For the EndoMT model, hREC were seeded at 380,000 cells per well in a 6-well plate or 190,000 cells per well in a 12-well plate. After 24 h of adherence, the medium was removed and replaced with complete medium containing a mixture of IL-1β (0.1 ng/mL), TNF-α (5.0 ng/mL) and TGF-β2 (5.0 ng/mL) (PeproTech, Rocky Hill, NJ, USA). At day three the medium was changed, and at day six, with the EndoMT induction considered complete, the cells were used for immunostaining and qPCR. After the six days of induction, the EndoMT phenotype was maintained with EBM basal medium containing 5% fetal bovine serum (FBS) plus 1% penicillin/streptomycin (17-602E, Lonza). The numbers of live cells were determined by hemocytometer counting following trypan blue staining.

For the prevention study using the p38 MAPK inhibitors, SB203580 or SB202190 (10 μM, Abcam, Cambridge, MA, USA) was applied with the pro-inflammatory cytokines at the beginning of induction and again at day three; expression of endothelial and EndoMT markers was analyzed by RT-qPCR at day six. For the intervention study, SB203580 was added at days six, nine and 12 post-induction, and gene expression was analyzed at day 15. To test responsiveness to VEGFA stimulation, the hREC and EndoMT cells were treated with 10 ng/mL of human vascular endothelial growth factor (VEGFA165) (#8065SF, Cell Signaling Technology, Danvers, MA, USA) for two hours at 37 °C in modified EGM-2 medium (without adding the VEGFA or hydrocortisone included in the bullet kit (Lonza)), then expression of VEGFA-induced genes (VCAM-1, E-Selectin, tissue factor) was evaluated by qPCR.

To evaluate the effects of high concentration of VEGFA in reversing EndoMT, VEGFA165 (50 ng/mL) was added to EndoMT cells for 7 days in basal EBM media containing 5% FBS and 1% penicillin/streptomycin (Lonza). All media was changed every 3 days.

### 2.2. Western Blot Assay

Cells were rinsed twice with ice-cold PBS, then cell lysates were harvested with extraction buffer (10 mM Tris-HCl, pH 7.4; 5 mM EDTA; 50 mM NaCl; 50 mM NaF; 1% Triton X-100; 20 μg/mL aprotinin; 2 mM Na_3_VO_4_; 1 mM phenylmethylsulfonyl fluoride; 2% sodium dodecyl sulfate (SDS)) prepared in protein sample buffer (Sigma-Aldrich). Cell lysate samples were centrifuged at 4 °C for 5 min at 13,000× *g*. The concentration of proteins in cell lysate was determined using the Micro BCA protein assay reagent kit (Pierce, Thermo Fisher Scientific, Rockford, IL, USA), following the manufacturer’s instructions. Cell lysates were boiled in loading buffer (500 mM dithiothreitol, 50% sucrose, 500 mM Tris HCl (pH 6.8), and 0.5% bromophenol blue) for 5 min. Total protein (15 μg) from each group was separated by 10% SDS-PAGE, transferred to polyvinylidene difluoride membranes, and subjected to western blot analyses with the appropriate primary and secondary antibodies (Supplementary Table S2). Experiments were repeated at least three times unless otherwise noted. The signal intensity was determined by densitometry using Image J software (https://imagej.nih.gov/ij/, NIH, Bethesda, MD, USA).

### 2.3. MTT Assay

To evaluate cell proliferation, equal numbers of live hREC and EndoMT cells were plated at 10,000 cells per well in a 48-well plate in 300 µL EBM-2 basal medium supplemented with 5% FBS and 1% penicillin/streptomycin (Lonza). After six hours of cell adhesion (time 0), cells were treated with medium containing or lacking 25 ng/mL VEGFA165 for 72 h. After the medium was removed, cells were washed once with PBS and incubated in 100 µL medium containing 1.2 mM MTT (3-(4,5-Dimethyl-2-thiazolyl)-2,5-diphenyl-2H-tetrazolium bromide; M2128, Sigma-Aldrich). Cells were incubated for 2 h at 37 °C, then 50 µL 100% DMSO was added to solubilize the MTT salt. The medium was homogenized and transferred to a 96-well plate, then absorbance (570 nm) was measured using a microplate reader (Synergy Mx; Biotek, Winooski, VT, USA). The data are expressed as fold changes between VEGFA165-stimulated cells and non-treated cells. All data were normalized to the baseline measurement at time 0.

### 2.4. Tube Formation Assay

To evaluation formation of tubes/cords, equal numbers of live hREC and EndoMT cells were plated at 90,000 cells per well in a 48-well plate coated with 140 µL polymerized collagen gel mixture, in 200 µL EBM-2 media supplemented with 5% FBS and 1% penicillin/streptomycin (Lonza). The collagen gel mixture was prepared using 0.5 mL 10× RPMI solution (31800022, Gibco, Thermo Fisher Scientific), 0.4 mL NaHCO_3_/NaOH solution (2 g of NaHCO_3_ plus 4 mL of 10 N NaOH diluted with distilled water to a final volume of 80 mL), 0.1 mL HEPES 1M solution (15630106, Gibco, Thermo Fisher Scientific), 2.5 µL fibronectin (F1141, Sigma-Aldrich), 2.5 µL laminin (L2020, Sigma-Aldrich), 4 mL bovine collagen solution (5005, Advanced BioMatrix, San Diego, CA, USA) and pH adjusted to 7.0 to 7.4. After 24 h, the medium was removed, 60 µL fresh collagen mixture was added to the top and allowed to polymerize (30 min at 37 °C), and 200 µL of EBM-2 basal media containing 0.5% horse serum (H0146, Sigma-Aldrich) and 0.1% bovine brain extract (CC-4098, Lonza), with or without VEGFA165 at 25 ng/mL, was added. Cells were incubated at 37 °C for five hours, images of cells were taken using 10× objective using the EVOS FL Auto microscope (Thermo Fisher Scientific, Waltham, MA, USA). Tube formation on images was quantified using the Angiogenesis Analyzer plugin for Image J software (NIH, https://imagej.nih.gov/ij/), with the experimenter masked to the treatment conditions.

### 2.5. Cell Migration Assay

To evaluate cell migration, equal numbers of live hREC and EndoMT cells were plated at 200,000 cells per well in a 24-well plate in EBM-2 basal media with 5% FBS, 1% penicillin/streptomycin (Lonza) for 24 h. Cells were then scratched using a p200 micropipette tip, washed three times with PBS, and provided with EBM-2 media with 5% FBS and 1% penicillin/streptomycin (Lonza) for baseline (time 0) photographs (10× objective, EVOS FL Auto microscope, Thermo Fisher Scientific). Fresh medium was added that contained or lacked 25 ng/mL of VEGFA165, and five hours later the cells were photographed again. The scratch areas were quantified using Adobe Photoshop (Adobe Systems, San Jose, CA, USA), with the experimenter masked to the treatment conditions. The data were expressed as wound closure ratio between the VEGFA165 stimulated cells and the corresponding non-stimulated cells, and normalized to the time 0 data.

### 2.6. Animal Studies

All animal procedures were reviewed and approved by the Committee for Ethics in Animal Research of the Schepens Eye Research Institute of Mass Eye and Ear (study protocol number: S-520-1021), and according to the Use of Animals in Ophthalmic and Vision Research (ARVO) Statement for the Use of Animals in Ophthalmic and Vision Research.

The JR5558 line of spontaneous CNV mice in the C57BL/6J background generated from an in-house colony, and age-matched wild-type C57BL/6J mice from the Jackson Laboratory (Bar Harbor, ME, USA), were used as previously described [19,21,22,23,24]. All animals received food and water ad libitum, with a 12-h day-night cycle and in a temperature-controlled environment. During all procedures, mice were anesthetized by intraperitoneal (IP) injection of a mixture of ketamine (100–120 mg/kg)/xylazine (20 mg/kg), and animals under anesthesia were kept warm using a heating blanket. Fluorescein fundus angiography (FA) was performed at P22–23 prior to selecting P24 pups for treatments. Only pups with at least five hyperfluorescent spots (leaky CNV lesions) in each eye based on the FA live imaging were used for the study. For FA, a drop of sterile saline was placed on the experimental eye to remove any debris before applying a contact lens. Next, 25% sodium fluorescein (pharmaceutical grade, Akorn Inc., Lake Forest, IL, USA) was injected intraperitoneally at 0.01 mL per 5 g of body weight. FA photos of the central retina (around the optic nerve) were taken sequentially at one, two, three and five minutes post fluorescein injection using the Spectralis^®^ equipped with a 55° lens in the scanning laser angiography mode (Heidelberg Engineering, Franklin, MA, USA). P24 mice were used for the treatment, since this is an established time point at which most of the CNV have already initiated and are undergoing active angiogenesis, and before the development of the severe retinal degeneration that is observed around P40 [21].

For treatment of JR5558 mice by intravitreal injection (IVT), P24 mice of both sexes were anesthetized by injection of ketamine and xylazine. The pupils were dilated using a drop of 1% Tropicamide (Bausch & Lomb, Tampa, FL, USA) followed by a drop of 0.5% Proparacaine (topical anesthetic; Bausch & Lomb) applied on the corneal surface. IVT injections were performed under a microsurgical microscope using a blunt 33-gauge needle. Diluted DMSO vehicle control, 5 nmoles of the p38MAPK inhibitor SB203580 (Abcam), 2.5 or 5.0 µg of anti-VEGFR2 neutralizing antibody (MAB4431; R&D Systems, Minneapolis, MN, USA), or a combination of SB203580 plus anti-VEGFR2 neutralizing antibody was injected into both eyes. Each treated eye was punctured at the upper nasal limbus and a volume of 1 μL was allowed to diffuse over ~15 s. All IVT dosing was done in a masked fashion. After IVT, the eyes were treated with a triple antibiotic (Neo/Poly/Bac) ointment (Bausch & Lomb). Only animals with no obvious signs of inflammation based on slit lamp examination at 24 to 48 h post injection were included in the study. Each litter of pups was divided into at least two different treatment groups plus control, to minimize the potential inter-litter variation of responses to the different treatments.

### 2.7. Real-Time Semi-Quantitative PCR (qPCR)

For in vitro and in vivo studies, total RNA was extracted from the cells or tissues (RPE/choroid complex) using the RNAeasy kit (Qiagen, Germantown, MD, USA), then the iSCRIPT kit (BioRad, Hercules, CA, USA) was used for cDNA synthesis per manufacturer’s instructions. Real-time qPCR was conducted using the Syber Green Gene Expression Assay (Roche Life Science, Indianapolis, IN, USA) using gene-specific primers pairs listed in Supplementary Table S1 [25,26,27,28,29,30,31,32,33,34]. Relative gene expression was determined using the ΔΔCt method after normalizing sample loading with the housekeeping gene HPRT1.

### 2.8. Histology and Immunostaining

For immunostaining of hREC and EndoMT cells, cells were fixed in 4% paraformaldehyde for 10 min at room temperature, washed with PBS, and blocked for one hour with 5% heat-inactivated FBS, and permeabilized in 0.5 to 0.3% Triton X-100 (Sigma-Aldrich) in PBS containing calcium and magnesium (14080-055, Gibco). The primary antibodies against αSMA; VE-cadherin, CD31, vimentin, SNAI1, fibronectin, as well as isotype-matched control IgGs and phalloidin (Supplementary Table S2) were diluted in blocking solution at and incubated with the cells overnight at 4 °C.

Eyes were fixed in 4% paraformaldehyde for 24 h at 4 °C, washed in phosphate buffered saline (PBS) and prepared as whole flat-mount eyecups (retina removed and RPE layer facing up) or transverse sections. For sections, eyes were cryoprotected in 30% sucrose, snap-frozen in optimum cutting temperature compound (Tissue-Tek, Sakura FineTek, Torrance, CA, USA), cut with a cryostat into 10 µm sections, and thaw-mounted onto glass slides. To quantify the CNV lesions using an established method [21,23], the eyes were dissected to isolate the eyecup containing the RPE/choroid tissue. Tissues were blocked for one hour with 10% heat-inactivated FBS and donkey serum (Jackson Immuno Research), then permeabilized in 0.5 to 0.3% Triton X-100 (wholemounts), and incubated with primary antibodies against αSMA; type III collagen; phospho-p38 MAPK, vimentin, SNAI1, fibronectin, NF-kB, IB4, rabbit IgG, mouse IgG Cy3, goat IgG and rat IgG (Supplementary Table S2) in blocking solution overnight at 4 °C. Secondary antibodies Alexa Fluor 647 donkey anti-rabbit IgG, Alexa Fluor 647 donkey anti-goat IgG and Alexa Fluor 594 donkey anti-goat IgG (Supplementary Table S2) were applied for one hour (cells or sections) or two hours (flat-mounts) at room temperature, together with the nuclear stain 4′,6-diamidino-2-phenylindole, dihydrochloride (DAPI) (D1306, Invitrogen, Thermo Fisher Scientific).

Fluorescence images were acquired with a Zeiss Axioscope 2 microscope fitted with a Zeiss AxioCam digital camera, using the Zeiss AxionVision software. Image stitching of flat-mounted whole eyecups (RPE facing up) was performed using Image J Fiji software (http://fiji.sc/, NIH). The numbers of CNV lesions per eyecup were visually counted, and CNV area per eyecup was determined based on the IB4-positive staining area using Adobe Photoshop (Adobe Systems), with values expressed in pixels. To quantify vimentin expression, we used values of luminosity, expressed in pixels and normalized to lesion area, from each individual CNV lesion. All images were quantified in a masked fashion.

### 2.9. Statistical Analysis

For real-time PCR analysis, each individual well for cell-based experiments was analyzed as an individual data point (*n*). For real-time PCR analysis using eye tissues, each individual eye was analyzed as an individual data point (*n*). Data from qPCR, cell proliferation (MTT assay), tube formation, and cell migration (scratch assay) were analyzed using 2-tail unpaired t-tests as indicated (GraphPad Prism, San Diego, CA, USA). For the JR5558 CNV model, each individual eye was analyzed as an individual data point (*n*) for the average CNV area and average number of CNV per eye, whereas the vimentin levels per individual CNV were analyzed as individual data points (*n*). The resulting data were analyzed using one-way ANOVA followed by a Dunnett’s post hoc test to compare the different test groups to the vehicle control, and also by 2-tail unpaired t-test to compare specific treatment groups as indicated (GraphPad Prism). For all comparisons, values of *p* < 0.05 were considered statistically significant. Data are shown as mean ± SEM unless otherwise noted. All animal experiments and all data analyses for both animal and cell-based experiments were performed in a masked fashion, and the minimum sample size was determined based on prior pilot experiments using the same models.

## 3. Results

### 3.1. Cytokine Treatment Induces EndoMT in Primary Human Endothelial Cells

To dissect the role of EndoMT in fibrosis associated with CNV pathogenesis, and to examine VEGFA signaling in endothelial cells undergoing EndoMT, we adopted a cell-based model. EndoMT was induced in primary human retinal endothelial cells (hREC) by treatment with the pro-inflammatory cytokines tumor necrosis factor α (TNF-α), interleukin 1β (IL-1β), and tumor growth factor β2 (TGF-β2), based on a model developed to study EndoMT of pulmonary endothelial cells [20]. This cell-based model of EndoMT is relevant to AMD because of the highly inflammatory microenvironment of the retina in this disorder [35].

**Figure 1:**
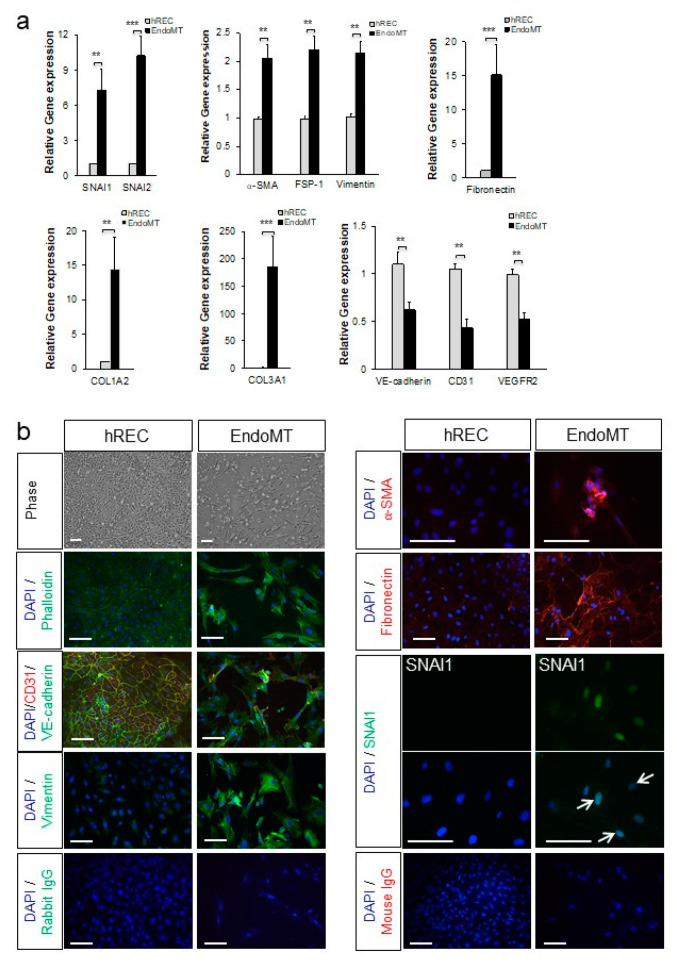
Primary human retinal endothelial cells (hREC) can be used to model endothelial-to-mesenchymal transition (EndoMT) in vitro. (**a**) Expression levels for the genes encoding the EndoMT-associated proteins SNAI1, SNAI2, α-SMA, FSP-1, vimentin, fibronectin, COL1A2, and COL3A1; and the endothelial differentiation markers VE-cadherin, CD31 and vascular endothelial growth factor receptor-2 (VEGFR2), are shown relative to the housekeeping gene HPRT1 and normalized to the control hREC as assessed by qPCR on day 6 of EndoMT induction. Data = mean ± SEM, ** *p* < 0.01, *** *p* < 0.001 compared to the control hREC by unpaired 2-tail t-test, *n* = 4 (VE-cadherin), 6 (collagen type III alpha 1 chain (COL3A1)), 8 (SNAI1, α-smooth muscle actin (α-SMA), fibroblast-specific protein 1 (FSP-1), CD31), 9 (vimentin), 10 (fibronectin, VEGFR2), and 11 (SNAI2, COL3A1) independent wells per group. (**b**) Histological analysis of EndoMT cells on day 6 of EndoMT induction. Phase contract microscopy and phalloidin staining (green) in the top four panels on the left illustrate the distinctions in cellular morphology between hREC and EndoMT cells. Alterations in expression and localization of endothelial differentiation markers CD31 and VE-cadherin and the mesenchymal markers vimentin, α-SMA, fibronectin and SNAI1 between hREC and EndoMT cells are also shown. Note the nuclear localization of SNAI1 (green, arrows) in EndoMT cells whereas the control hREC do not display SNAI1 expression. Isotype-matched IgG staining controls are shown on the bottom rows. Scale bars = 100 µm.

After determining the optimal cytokine dose (0.1 ng/mL IL-1β, 5.0 ng/mL TNF-α, and 5.0 ng/mL TGF-β2), and the optimal time point for evaluation of gene expression (day six after initial cytokine treatment, with cytokines reapplied at day three) (Supplementary Figure S1), expression of genes associated with EndoMT and endothelial cell differentiation was evaluated. Treatment of hREC with the optimal doses of TNF-α, IL-1β, and TGF-β2 significantly increased expression of a variety of EndoMT-associated genes, including those encoding snail family transcriptional repressors 1 and 2 (SNAI1 and SNAI2, considered the master regulators of EndoMT and fibrosis [36,37]), α-SMA, fibroblast-specific protein 1 (FSP-1), vimentin, fibronectin, collagen type I alpha 2 chain (COL1A2) and collagen type III alpha 1 chain (COL3A1), compared to untreated endothelial cells (*p* < 0.01 to *p* < 0.001, Figure 1a). Conversely, expression levels for genes encoding VE-cadherin, CD31, and VEGFR2, markers of differentiated vascular endothelial cells, were significantly reduced compared to the untreated hREC control (*p* < 0.01). The increases in expression of most genes associated with EndoMT, and decreases in expression of those associated with endothelial differentiation, persisted for nine days (SNAI2, FSP-1, vimentin, fibronectin, COL1A2, COL3A, CD31) and even for up to 15 days (VEGR2, VE-cadherin) post-induction in normal culture conditions (i.e., without the pro-inflammatory cytokines;  Supplementary Figure S2a).

The EndoMT phenotype was confirmed at the protein level by immunostaining and microscopy. As shown in Figure 1b, the cytokine-treated hREC adopted a fibroblast-like morphology by day six post-induction. Stress fibers (phalloidin staining) were prominent, and VE-cadherin, an endothelial cell-cell junction protein, was nearly absent. In addition, staining for markers of the mesenchymal phenotype, namely vimentin, α-SMA, fibronectin, and nuclear localization of SNAI1, were visible in the cytokine-treated hREC. The cell death resulting from the cytokine treatment would be expected to induce EndoMT in this cell-based model, as EndoMT is an adaptive response to cellular stress for EC [20]. Protein expression and localization patterns associated with EndoMT (α-SMA, vimentin, COL3A, VE-cadherin, and CD31) persisted for 15 days (Supplementary Figure S2b). Image intensity analysis of the imunostaining and western blot analysis confirmed a significant increase in expression of EndoMT markers and significant reduction in expression of differentiated endothelial cell markers in the EndoMT cells compared to hREC (Supplementary Figure S3). 

Our data show that gene expression profiles and protein localization consistent with EndoMT can be induced and maintained in hREC, providing a robust cell-based model to study EndoMT of the retinal vasculature. The cytokine-treated hREC are therefore referred to hereafter as EndoMT cells.

### 3.2. VEGFA Reverses EndoMT in Primary Human Endothelial Cells

**Figure 2:**
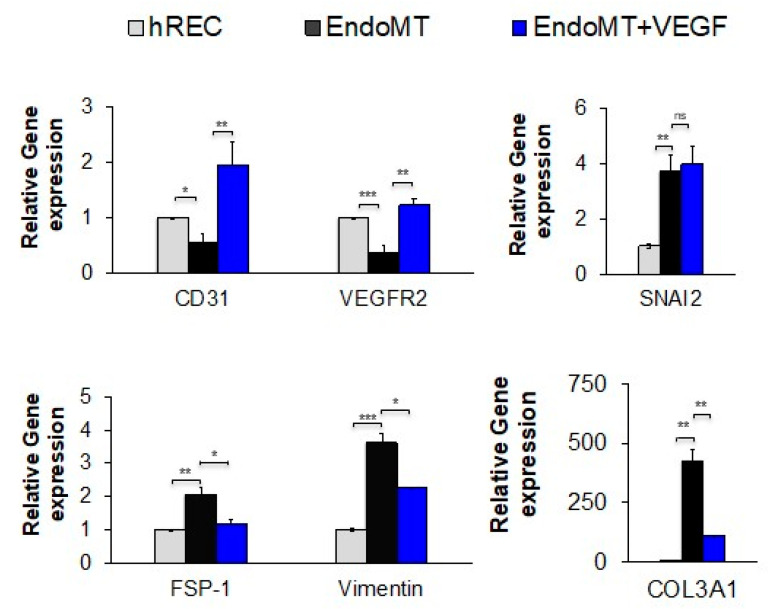
Treatment with VEGFA at a high concentration significantly reduces expression of genes associated with EndoMT and restores gene expression associated with endothelial cell differentiation. After EndoMT induction using IL-1β (0.1 ng/mL), TNF-α (5.0 ng/mL), and TGF-β2 (5.0 ng/mL), EndoMT cells were cultured for seven days in medium containing 5% FBS and 1% penicillin/streptomycin in the presence or absence of VEGFA165 (50 ng/mL). Expression levels for the genes encoding the EndoMT-associated proteins SNAI2, FSP-1, vimentin, and COL3A1, as well as the endothelial differentiation markers CD31 and VEGFR2, are normalized to the housekeeping gene HPRT1 and shown relative to the hREC control as assessed by qPCR. (*n* = 3 independent wells per group). Data = mean ± SEM. * *p* < 0.05, ** *p* < 0.01, *** *p* < 0.001, 2-tail unpaired t-test.

During the pathogenesis of wet AMD, high levels of VEGFA promote CNV and might also suppress EndoMT to allow for efficient neovascularization [16,18]. To determine the effects of VEGFA on EndoMT, EndoMT cells were treated with VEGFA for six days following EndoMT induction, in the absence of pro-inflammatory cytokines, then gene expression was evaluated (Figure 2). This treatment condition models the return to high levels of intraocular VEGFA after a bolus injection of a VEGFA antagonist, as occurs in clinical treatment of CNV [38]. The genes selected for analysis were those that we found to be expressed or suppressed for at least nine days after induction of EndoMT (Supplementary Figure S2).

Treatment for six days with high levels of VEGFA protein (50 ng/mL) restored expression of VEGFR2 and induced higher levels of CD31 expression in EndoMT cells compared to the untreated EndoMT cells (Figure 2 first panel), demonstrating a return to endothelial cell differentiation. Compared to untreated EndoMT cells, VEGFA treatment significantly suppressed expression of the EndoMT markers FSP-1, vimentin and COL3A1, though it had no effect on expression of SNAI2 (Figure 2, remaining panels). These data suggest that high levels of VEGFA alone may not be sufficient to completely reverse EndoMT.

### 3.3. Inhibition of p38 MAPK Activation Prevents and Reverses Changes Associated with EndoMT in hREC

Since the p38 MAPK pathway is known to participate in the SNAI1-induced general fibrotic pathway, which includes EndoMT [39,40,41], and since we observed an increase in phosphorylated p38 MAPK during EndoMT induction of hREC (Supplementary Figure S4), as well as phosphorylated p38 MAPK expression was associated with the spontaneous CNV in the JR5558 mouse (Figure 5e), we next explored the effects of two small-molecule inhibitors of p38 MAPK using the hREC EndoMT model. The inhibitors SB203580 and SB202190 (both are p38α- and p38β2-selective inhibitors) have specificities and potencies against different MAPK family members, namely p38α, p38β, p38γ and p38δ [42,43], and were added either together with the cytokines used to induce EndoMT (prevention study) or six days after induction of EndoMT (intervention study). Both drugs were used at 10 µM final concentration based on a pilot dose escalation study in which this dose and a higher dose (20 µM) yielded similar levels of EndoMT suppression and expression of markers associated with differentiated endothelial cells (Supplementary Figure S5).

**Figure 3:**
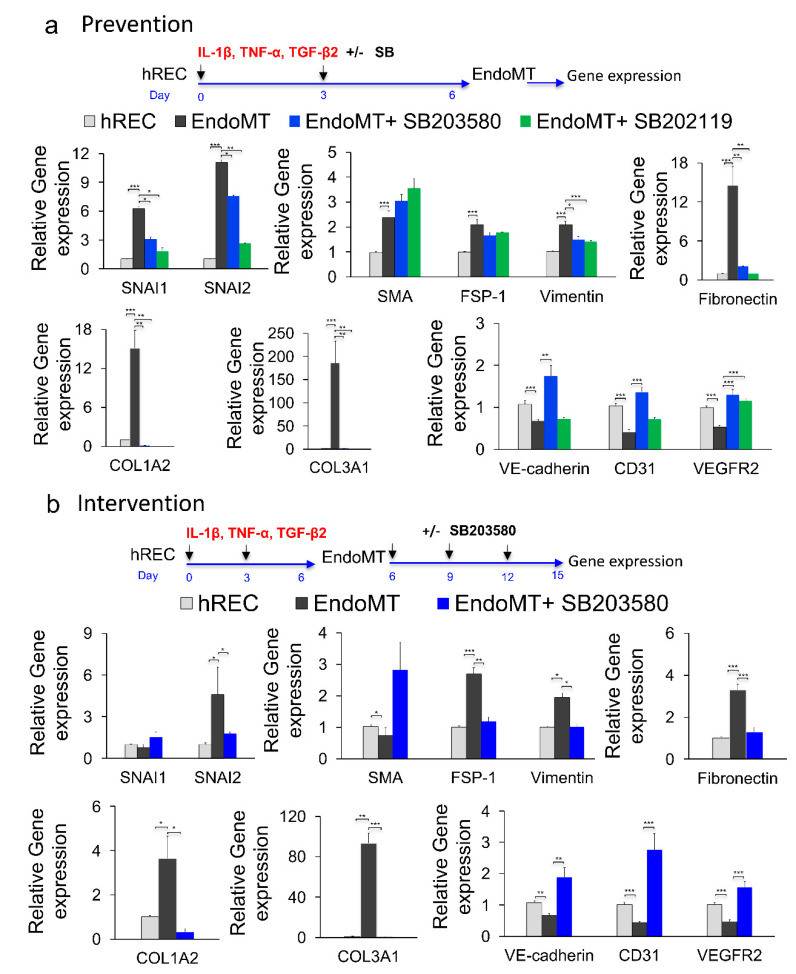
Phospho-p38 MAPK inhibitors prevent and reverse EndoMT-associated gene expression in vitro. (**a**) To study possible prevention of EndoMT, hREC were treated with the p38 MAPK inhibitor SB203580 or SB202190 (10 μM) during EndoMT induction. Expression levels for the genes encoding the EndoMT-associated proteins SNAI1, SNAI2, α-SMA, FSP-1, vimentin, fibronectin, COL1A2 and COL3A1; and the endothelial differentiation markers VE-cadherin, CD31 and VEGFR2 were assessed by qPCR on day 6 of EndoMT induction. Data = mean ± SEM, * *p* < 0.05, ** *p* < 0.01, *** *p* < 0.001 by 2-tail unpaired t-test relative to the untreated EndoMT controls, *n* = 7 (COL3A1, VE-cadherin), 9 (α-SMA, FSP-1, vimentin), 10 (SNAI1, SNAI2, fibronectin, COL1A2, CD31), 12 (VEGFR2) independent wells. (**b**) To study reversal of EndoMT, EndoMT cells were treated with SB203580 (10 μM) for 9 days after EndoMT induction. Expression levels for the genes encoding EndoMT-associated proteins, and endothelial differentiation markers were assessed by qPCR after 9 days of treatment. Data = mean ± SEM, * *p* < 0.05, ** *p* < 0.01, *** *p* < 0.001 by 2-tail unpaired t-test, relative to the untreated EndoMT controls; *n* = 3 (COL1A2) and *n* = 6 (the rest of the genes) independent wells.

In the prevention study, both SB203580 and SB202190 suppressed cytokine-induced expression of the EndoMT-associated genes encoding SNAI1, SNAI2, vimentin, fibronectin, COL1A2, and COL3A1 (*p* < 0.05 to *p* < 0.001, Figure 3a), though neither prevented the increase in expression of the genes encoding α-SMA and FSP-1. However, only SB203580 preserved expression of the full set of endothelial differentiation markers (*p* < 0.01 to *p* < 0.001 for the increase in expression of CD31, VE-cadherin, and VEGFR2 relative to EndoMT); SB202190 preserved expression only of VEGFR2 (*p* < 0.001 compared to EndoMT). This difference led to the selection of SB203580 as the sole EndoMT inhibitor for subsequent studies.

Treatment with SB203580 from day six to 15 post-induction of EndoMT (intervention study) decreased expression of the genes encoding FSP-1, vimentin, SNAI2, fibronectin, COL1A2, and COL3A1 (*p* < 0.05 to *p* < 0.001) while inducing expression of the gene encoding α-SMA (*p* < 0.05) and not affecting expression of the gene encoding SNAI1 (Figure 3b). Importantly, SB203580 increased expression of all three markers of endothelial cell differentiation (CD31, VE-cadherin and VEGFR2; *p* < 0.01 to *p* < 0.001). Note that expression of SNAI1 and α-SMA by EndoMT cells in the intervention study (day 15) was not increased compared to the control hREC, this is due to discontinued pro-inflammatory cytokines supplementation for an additional nine days of culture in this experiment. These data support use of SB203580 as an experimental drug for inhibiting EndoMT in this study. However, it is important to note that SB203580 did not fully prevent EndoMT nor fully reverse the EndoMT phenotypes in this model, likely because EndoMT is mediated by more than one signaling pathway.

### 3.4. The EndoMT Inhibitor SB203580 Restores VEGFA Responsiveness in EndoMT Cells

**Figure 4:**
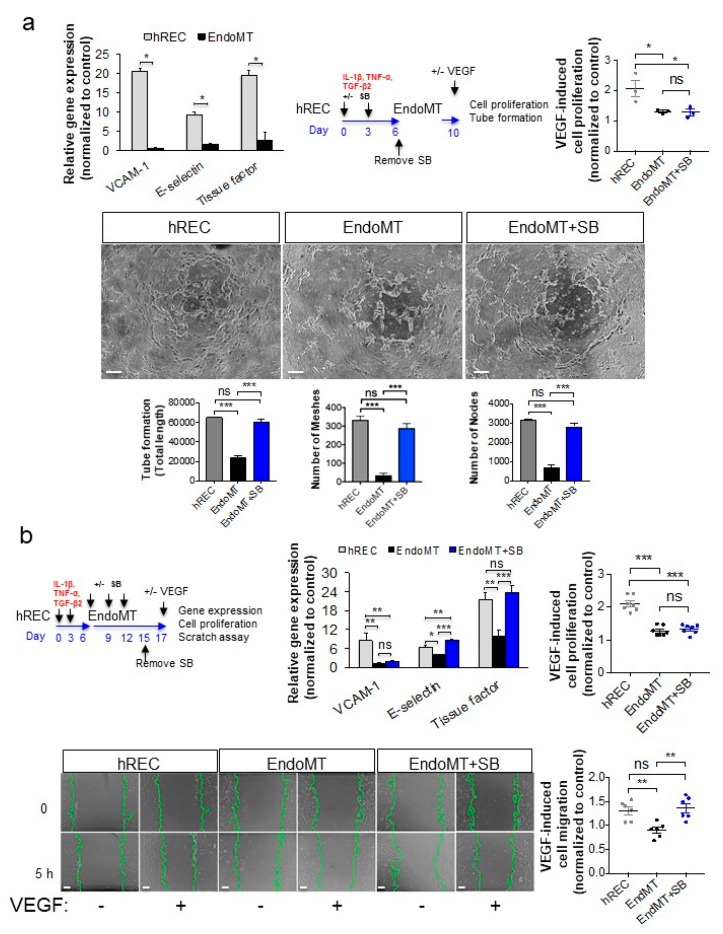
Treatment with an EndoMT inhibitor significantly restores VEGFA-mediated gene expression and endothelial cell functions in EndoMT cells. (**a**) To assess responsiveness to VEGFA, hREC, and EndoMT cells were treated with hVEGFA165 (10 ng/mL) for 2 h, then expression of genes encoding VCAM-1, E-selectin and TF was assessed by qPCR (top left panel). For the prevention study, cells were treated with SB203580 (10 μM) during EndoMT induction, then four days later hVEGFA165 (10 ng/mL) was added. Cell proliferation was assessed using the MTT assay (top right panel), and VEGFA-induced cord/tube formation was assessed using a collagen gel assay (bottom panels). Equal numbers of viable hREC and EndoMT cells were used for these assays. Data = mean ± SEM, * *p* < 0.05, ** *p* < 0.01, *** *p* < 0.001 by 2-tail unpaired t-test, *n* = 6 independent wells for the first panel and *n* = 3 independent wells for the subsequent panels. In the micrographs, scale bar = 100 μm. (**b**) For the intervention study, EndoMT cells were treated with SB203580 (10 μM) for 9 days, starting 6 days after induction of EndoMT, then treated with hVEGFA165 (10 ng/mL; top left panel). Expression of the VEGFA-induced genes encoding VCAM-1, E-selectin and TF was assessed by qPCR (top middle panel), cell proliferation was determined using the MTT assay (top right panel), and cell migration was assessed using a scratch assay (bottom panels). Equal numbers of viable hREC and EndoMT cells were used for these assays. Scale bars = 100 μm. Data = mean ± SEM, * *p* < 0.05, ** *p* < 0.01, *** *p* < 0.001 by 2-tail unpaired t-test. *n* = 8 independent wells for the gene expression assay, 7 independent wells for the MTT assay, and 6 independent wells for the scratch assay. All image analysis was performed by a trained investigator who was masked to the identity of the treatment groups.

Because SB203580 suppresses EndoMT and re-established expression of the endothelial markers tested, including VEGFR2, we examined whether SB203580 could restore endothelial functions associated with responsiveness to VEGFA stimulation, namely gene expression, cell proliferation, cell migration, and tube/cord formation. In the absence of SB203580 the EndoMT cells treated with VEGFA showed markedly reduced expression of the VEGFA-induced genes encoding VCAM-1, E-selectin and TF after two hours of VEGFA stimulation compared to hREC control (*p* < 0.05, Figure 4a). These genes were selected for evaluating the responsiveness to VEGFA because they are robustly induced by VEGFA treatment in endothelial cells [44,45,46,47]. The EndoMT cells also displayed reduced cell proliferation and tube/cord formation in response to VEGFA, relative to the hREC controls that were plated at the same density (*p* < 0.05 and *p* < 0.001, respectively, Figure 4a). Addition of SB203580 from day 0 until day six (prevention study) failed to restore VEGFA-induced proliferation of EndoMT cells at day 10, but the inhibitor did significantly increase VEGFA-induced endothelial tube/cord formation, to levels comparable with those of the control hREC cells (*p* < 0.001, Figure 4a, bottom graphs).

We next tested the effectiveness of SB203580 in re-establishing VEGFA responsiveness in an interventional study. After induction of EndoMT by treating hREC with pro-inflammatory cytokines for six days, the resulting EndoMT cells were treated with SB203580 for an additional nine days, after which the cells were exposed to VEGFA. Treatment with SB203580 significantly increased VEGFA-induced expression of E-selectin and TF (*p* < 0.001), though not VCAM-1, in EndoMT cells (Figure 4b). VEGFA-induced cell proliferation was not returned, but VEGFA-induced cell migration in a scratch assay was significantly increased compared to the untreated EndoMT control (*p* < 0.01), to a level comparable to the hREC (Figure 4b, bottom graph). The scratch assay was used as an additional functional assay for the intervention study because the long duration of culture was not compatible with the VEGFA-induced tube/cord formation assay. These data support evaluation of SB203580 for reducing and/or reversing EndoMT in an animal model.

### 3.5. EndoMT and Fibrosis Occur During Establishment of Spontaneous CNV Lesions in a Mouse Model

To examine the role of EndoMT and EndoMT reversal in CNV in vivo, we employed the JR5558 model of spontaneous CNV. This model is uniquely suited for studying fibrotic changes associated with CNV since, unlike the laser-induced CNV model, the CNV lesions develop with age and continue to grow without regressing, resulting in multiple CNV lesions with fibrotic changes. Furthermore, the established CNV lesions in older mice can become fibrovascular membrane-like structures consistent with those observed in human AMD. Finally, the CNV lesions in the JR5558 mice have a highly pro-inflammatory microenvironment (Supplementary Figure S6) similar to that observed in patients with wet AMD [35,48]. Since C57BL6/J control mice do not develop any spontaneous CNV, only the JR5558 mice were used to illustrate the progression of EndoMT/fibrosis with age in the following experiments.

**Figure 5:**
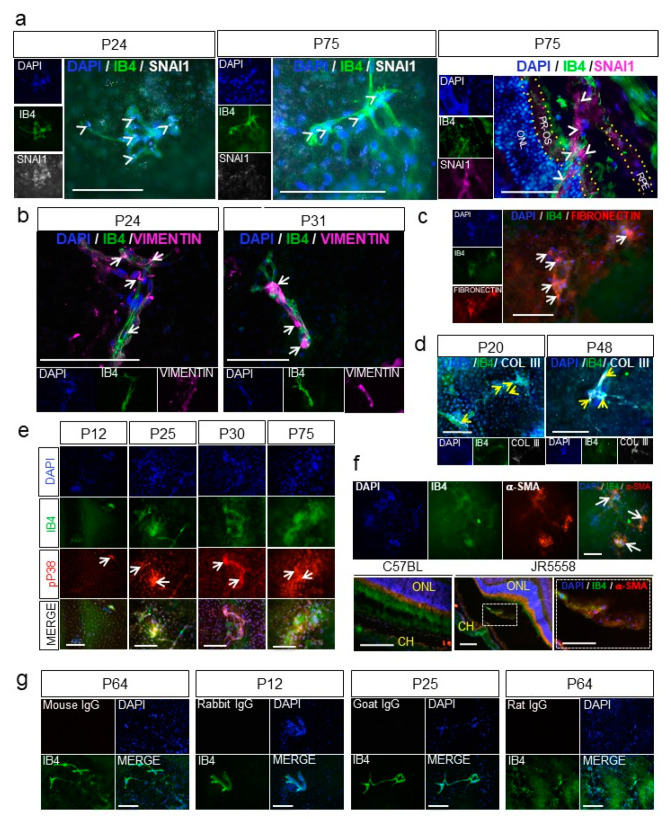
EndoMT and fibrosis are associated with spontaneous choroidal neovascularization (CNV) in a mouse model of wet age-related macular degeneration (AMD). (**a**) Nuclear expression of the zinc-finger transcription factor SNAI1 (white, arrowheads) by the endothelial cells of the CNV (IB4, green) was visualized by immunostaining flat-mount whole retinal pigment epithelium (RPE)–choroid eyecups from JR5558 mice at P24 (left panels) and P75 (middle panels). Cross sections of the RPE–choroid showing the CNV at P75 (arrowheads, right panel) were also immunostained for SNAI1 (purple). ONL, outer nuclear layer; PR-OS, photoreceptor outer segment; RPE, retinal pigment epithelium are outlined by yellow dotted lines. (**b**) Expression of vimentin (purple) in the vessels of the CNV (IB4, green) was visualized by immunostaining flat-mount whole RPE–choroid eyecups from JR5558 mice at P24 and P31. Note the increase in vascular staining of vimentin in the CNV at P31 compared to P24. (**c**) Colocalization of fibronectin expression (red) and the vessels of the CNV (IB4, green, arrows) was visualized by immunostaining flat-mount whole RPE–choroid eyecups of JR5558 mice at P70. (**d**) Collagen type III expression (white, yellow arrows) was visualized in developing CNV in flat-mount whole RPE–choroid eyecups from JR5558 mice at P20 and P48. (**e**) Co-localization of phospho-p38 MAPK (pP38, red) expression with the vessels of the CNV lesions (IB4, green arrows) was visualized by immunostaining flat-mount whole RPE–choroid eyecups from JR5558 mice at ages P12 to P75. (**f**) Expression of α-SMA (red) was visualized in vessels of CNV lesions (isolectin B4, green) by immunostaining flat-mount whole RPE–choroid eyecup tissues from P70 JR5558 mice (top panels), and histological sections of RPE–choroid tissues from P70 wild-type C57BL/6J and JR5558 mice (bottom panels). Co-localization of α-SMA with the CNV is shown with the arrows; the area of higher magnification in the cross section of the CNV lesion is indicated by the dotted lines. ONL, outer nuclear layer; CH, choroid layer. (**g**) Appropriate IgG controls were used for immunostaining at various time points. In micrographs of all panels, scale bars = 100 µm.

Immunostaining of the flat-mount RPE–choroid eyecup tissues (retina removed, en face view of the RPE layer) showed that SNAI1, the master transcription factor for EndoMT, was expressed by many endothelial cells in the CNV (on top of the RPE layer) in both the early (P24) and established (P75) lesions (Figure 5a, first two panels, arrowheads). In a cross section of the RPE–choroid layers of established CNV (P75), SNAI1 was also readily detected in the CNV positive for the endothelial cell marker isolectin B4 (IB4) (Figure 5a, right panel arrowheads). Expression and localization of vimentin, fibronectin and Col III as markers of EndoMT were also examined. Vimentin staining was observed at IB4-positive vessels of the CNV, with more robust staining observed in older mice as the lesions became more established (Figure 5b and Supplementary Figure S7). Strong staining for fibronectin (Figure 5c) and Col III (Figure 5d) were also associated with the CNV lesions, with staining persistent and even more prominent in the older, more established CNV.

Increased activation of p38 MAPK is also strongly associated with EndoMT and fibrosis during wound healing [49]. To determine whether p38 MAPK was activated in the CNV lesions in the JR5558 mouse model, flat-mount RPE–choroid eyecups were immunostained for phosphorylated p38 MAPK. Strong staining was detected at the CNV lesions, co-localized with the IB4-positive CNV vessels, and became more striking with increasing age of the mice (Figure 5e). The blood vessels of CNV on flat-mount eyecups of P70 JR5558 mice were also positive for α-SMA, but with a patchy and uneven staining (Figure 5f, top row, arrows) that is similar to the α-SMA staining found in pathological angiogenesis [50,51]. Immunohistological staining of eye sections showed that α-SMA-positive cells were closely associated with CNV and also occasionally co-localized with IB4-positive cells of the CNV in the subretinal space (bottom row), consistent with EndoMT in the CNV lesions. Staining levels for α-SMA became more prominent with age (i.e., progression) of the CNV (Supplementary Figure S7), consistent with development of fibrosis.

**Figure 6:**
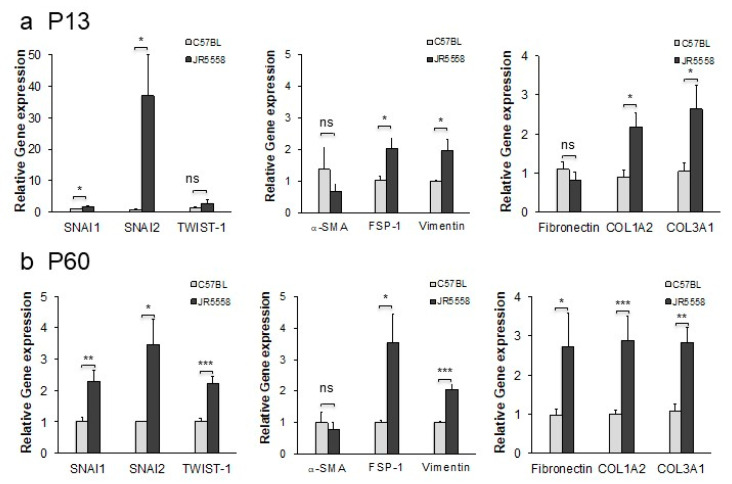
Expression of marker genes for EndoMT in the RPE–choroid of mice with spontaneous CNV is elevated relative to wild-type controls at both P13 and P60. (**a**) Expression levels for the genes encoding the EndoMT-associated proteins SNAI1, SNAI2, TWIST-1, α-SMA, FSP-1, vimentin, fibronectin, COL1A2, and COL3A1 were assessed by qPCR using mRNA isolated from RPE–choroid complexes from P13 JR5558 and age-matched C57BL/6J mice. (**b**) Expression of EndoMT-associated genes in RPE–choroid complexes from P60 JR5558 and age-matched C57BL/6J mice. Data = mean ± SEM, * *p* < 0.05, ** *p* < 0.01, *** *p* < 0.001 compared to the C57BL/6J mice as controls by unpaired 2-tail t-test, *n* = three mice (6 eyes) per group.

To validate the observation that expression of proteins associated with EndoMT is associated with progression of CNV, mRNA was extracted from the RPE/choroid complex, which contains the CNV lesions, and levels of EndoMT markers were evaluated by semi-quantitative real-time PCR. The genes encoding the EndoMT-associated transcription factors SNAI1 and SNAI2 were expressed at significantly higher levels in the RPE/choroid complex from the JR5558 mice at P13, when CNV lesions are starting to form, compared to wild-type controls (*p* < 0.05, Figure 6a). Genes encoding additional EndoMT markers, namely FSP-1, vimentin, COL1A2, and COL3A1, were also expressed at significantly higher levels in the JR5558 mice compared to wild-type controls (*p* < 0.05). The RPE/choroid complex containing older, more-established CNV (P60) yielded significantly higher levels of mRNA encoding SNAI1, SNAI2, TWIST-1, FSP-1, vimentin, fibronectin, COL1A2, and COL3A1 compared to wild-type controls (*p* < 0.05 to *p* < 0.001); levels of α-SMA mRNA were not significantly different (Figure 6b), consistent with the patchy staining data noted above. Together, these data suggest that persistent and often progressive EndoMT and fibrosis are associated with CNV growth in the JR5558 mouse. It is important to note that the real-time PCR data cannot distinguish EndoMT from epithelial–mesenchymal transition (EMT), and that EMT of RPE/choroid together with EndoMT likely contribute to the fibrotic changes detected in Figure 6.

### 3.6. Inhibition of VEGFR2 Exacerbates EndoMT, and SB203580 Suppresses EndoMT Induced by VEGFA Pathway Antagonism

Since we hypothesize that the low or reduced clinical efficacy observed with anti-VEGFA therapies could be due to EndoMT naturally occurring with CNV and/or induced by the VEGFA-targeting therapies, we examined EndoMT and fibrosis in the CNV of mice treated with anti-VEGFR2 function-neutralizing antibody. We have previously shown that this VEGFA pathway inhibitor can reduce CNV area by up to ~50% when provided at a high dose in the JR5558 mice [19,21,23].

**Figure 7:**
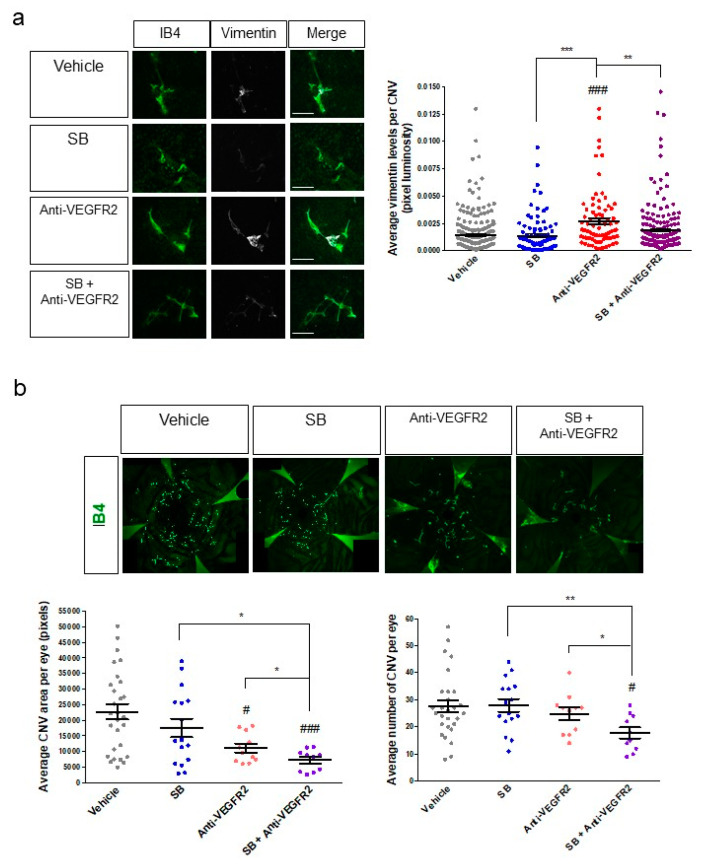
Inhibition of VEGFR2 as a solo treatment promotes EndoMT, whereas anti-VEGFR2 therapy in combination with inhibition of EndoMT/fibrosis reduces CNV area and CNV number in a mouse model of wet AMD. (**a**) Eyes of P24 JR5558 mice were treated with a single intravitreal injection of vehicle, SB203580 (10 nmoles), anti-VEGFR2 neutralizing antibody (5 μg), or a combination of SB203580 and anti-VEGFR2 antibody. RPE–choroid eyecups with retina removed were harvested seven days post-injection, prepared as flat-mounts, and immunostained for the EndoMT marker vimentin (white) in addition to the vascular marker IB4 (green) in the CNV (left panels). Luminosity of the vimentin staining was quantified and normalized to CNV lesion area (right panel). (**b**) Eyes of P24 JR5558 mice were treated with a single intravitreal injection of vehicle, SB203580 (10 nmoles), anti-VEGFR2 neutralizing antibody (2.5 μg), or a combination of SB203580 and anti-VEGFR2 antibody. RPE–choroid eyecups were harvested seven days post-injection, prepared as flat-mounts, and immunostained for isolectin B4 (IB4, green) to visualize the vasculature in the CNV (top panels). Micrographs of the IB4-stained eyecups were used to quantify total CNV area per eye and the number of CNV lesions per eye (bottom panels). For micrographs in A, scale bars = 100 μm. For graphs, data = mean ± SEM. ^#^ *p* < 0.05, ^###^ *p* < 0.001 by one-way ANOVA with Bunnett’s multiple comparison test compared to the vehicle control. * *p* < 0.05, ** *p* < 0.01, *** *p* < 0.001 by 2-tail unpaired *t*-test. *n* = 240 (Vehicle), 95 (SB), 83 (Anti-VEGFR2), and 186 (SB+Anti-VEGFR2) individual CNV per group in (a), and 28 (Vehicle), 16 (SB), 11 (Anti-VEGFR2), and 10 (SB+Anti-VEGFR2) individual eyes per group in (**b**). All dosing in animals was performed in a masked fashion and all image analysis was performed by a trained investigator who was masked to the identity of the treatment groups.

CNV lesions of the mice injected with anti-VEGFR2 antibody were immunostained for the EndoMT marker vimentin, shown above to be consistently associated with EndoMT, with expression levels generally correlating with progression of EndoMT and fibrosis in both the cell-based model and the JR5558 mice. A significant increase in CNV-associated vimentin expression was observed following injection of anti-VEGFR2 compared to the vehicle control (*p* < 0.001, Figure 7a), suggesting that inhibition of VEGFR2 signaling can promote EndoMT and fibrosis in CNV.

Since SB203580 suppressed aspects of EndoMT in our cell-based model, we injected this inhibitor into the eyes of JR5558 mice. Injection of SB203580 alone did not significantly reduce the baseline expression of vimentin compared to the vehicle control (Figure 7a), suggesting that it may not independently reduce spontaneous development of baseline EndoMT of the CNV described above. However, when SB203580 was co-injected with anti-VEGFR2 in the JR5558 mice, the increase in vimentin expression induced by anti-VEGFR2 was fully suppressed (*p* < 0.01 compared to anti-VEGFR2 alone, Figure 7a), suggesting that SB203580 might enhance the efficacy of VEGFA-targeting therapies for CNV by reducing the EndoMT induced by VEGFA pathway antagonism.

### 3.7. The EndoMT/Fibrosis Inhibitor SB203580 Enhances the Therapeutic Efficacy of VEGFR2 Antagonism for CNV in a Mouse Model

To test the effect of co-injection of SB203580 and anti-VEGFR2 on CNV outcomes, CNV area, and number of CNV lesions were assessed. A single intravitreal injection of SB203580 alone at 5 nmoles per eye did not significantly affect CNV area or number per eye compared to vehicle control (Figure 7b), consistent with our observations above showing that baseline EndoMT/fibrosis of CNV is not affected by this treatment. A single intravitreal injection of anti-VEGFR2 neutralizing antibody significantly reduced the average area of CNV by about 50% per eye compared to the vehicle control by seven days post-injection (*p* < 0.05), without reducing the number of CNV lesions (Figure 7b). These results were expected, since VEGFR2 antagonism reduces the growth of CNV but is not known to cause significant regression of CNV lesions that are already present. Combination treatment with anti-VEGFR2 and SB203580 reduced CNV area by about 70% and lesion number per eye by about 36% compared to vehicle control (*p* < 0.001 and *p* < 0.05, respectively, Figure 7b), a significantly improvement in both CNV area and lesion number compared to treatment with anti-VEGFR2 alone (*p* < 0.05 for both, Figure 7b). These data suggest that the combination of VEGFA pathway inhibition and EndoMT/fibrosis inhibition could further suppress CNV growth and might even cause regression of CNV.

## 4. Discussion

Poor responsiveness to anti-VEGFA therapies is a critical obstacle in the treatment of late-stage AMD. The development of resistance to anti-VEGFA therapy in patients who initially responded well suggests that other pathway(s) involved in CNV pathogenesis is (are) playing a role. Our data support a role for EndoMT in CNV and suggest that this process can negatively affect the efficacy of anti-VEGFA therapy. EndoMT in CNV is likely a survival mechanism for endothelial cells under stress in the normally avascular environment of the sub-retinal space, which likely lacks the optimal levels of growth factors such as VEGFA that are needed for vascular survival and homeostasis. EndoMT would be exacerbated by pro-inflammatory cytokines and the presence of TGF-β, and in fact the microenvironment of spontaneous CNV in the JR5558 mouse is highly pro-inflammatory, which would promote EndoMT of the CNV. We were able to confirm a role of VEGFA at high concentration in reversing some aspects of EndoMT, by suppressing the expression of many of the EndoMT markers and inducing the expression of markers for differentiated endothelial cells including VEGFR2. These in vitro data support the in vivo observation that high levels of VEGFA during CNV can inhibit EndoMT [16] and further apply this to the context of pathological angiogenesis in the eye. Importantly, blocking the VEGFA pathway significantly promoted EndoMT in CNV. Since EndoMT reduces the responsiveness of endothelial cells to VEGFA, this would negatively affect the therapeutic efficacy of VEGFA pathway inhibition. This observation in our animal model parallels the observation in clinical trials that dose-escalation of anti-VEGFA therapeutics consistently failed to yield the expected dose-dependent therapeutic effect for wet AMD [4,5]. Both the lack of a dose–response effect and the development of resistance to anti-VEGFA in patients after repeated treatments could be caused by the development of EndoMT of the CNV resulting at least partly from the anti-VEGFA therapy.

The in vitro EndoMT assay with hREC showed that the EndoMT phenotype is long-lasting and reversible, in good agreement with EndoMT being an adaptive response to promote endothelial cell survival in stressful environment. The hREC EndoMT assay was invaluable in identifying a small-molecule inhibitor that could prevent and reverse EndoMT. That a p38 MAPK inhibitor, SB203580, was effective is not surprising given the central role of this pathway in inflammation and TGF-β signaling in endothelial cells [43]. We believe that the hREC EndoMT assay will be useful in identifying more specific and likely more potent EndoMT inhibitors, for example by targeting a central transcription factor for EndoMT such as SNAI1-2 or ZEB1-2. Alternate inhibitors of EndoMT might also improve the therapeutic efficacy of anti-VEGFA therapy for CNV, and will be important to explore, as the p38 MAPK inhibitors reported here might lack specificity for EndoMT and/or CNV in the ocular environment, which is in good agreement with our finding that SB203580 failed to inhibit the background levels of EndoMT of the CNV. Since EndoMT is critical for normal development, e.g., of the heart, it will be important to choose inhibitors that limit potential off-target effects, allowing efficacious and safe treatment for CNV associated with end-stage AMD.

Perhaps most importantly, the small-molecule inhibitor SB203580 could suppress EndoMT induced by VEGFR2 inhibition and enhance the therapeutic efficacy of VEGFA pathway blockade for CNV. A recent study examining EndoMT in tumor vessels associated with glioblastoma showed that activation of the PDGF pathway contributes to development of EndoMT and resistance to anti-VEGFA therapy [15]. Although PDGF does activate p38 MAPK [43], EndoMT in CNV more likely involves local inflammation, TGF-β signaling and depletion of VEGFA rather than PDGF signaling. The glioblastoma findings support our observations regarding the relevance of EndoMT and possible mechanism for resistance to anti-VEGFA in pathologic neovascularization, and suggest further exploration of combination therapy with novel EndoMT inhibitors and anti-VEGFA in neovascular disorders.

Only the combination treatment with anti-VEGFR2 and the EndoMT/fibrosis inhibitor SB203580 resulted in a significant reduction in the number (as opposed to area) of spontaneous CNV lesions in the JR5558 mouse. This result suggests that regression of existing CNV occurred, as the majority of CNV lesions form before P24, the age at which the treatment started [21]. In clinical practice anti-VEGFA therapies alone fail to significantly regress CNV; instead, only mild pruning of small vessels and inhibition of vascular leakiness are typically observed in the CNV [3]. This suggests that CNV vessels may be largely independent of VEGFA for survival, potentially due, at least in part, to EndoMT. We believe that targeting EndoMT in combination with anti-VEGFA in the clinical setting could yield significant regression of CNV, translating to better short- and long-term therapeutic effects.

Lastly, while our results highlight the dramatic reduction in responsiveness to VEGFA displayed by EndoMT cells, EndoMT cells have been shown to have heightened responsiveness to other growth factors that are more specific for mesenchymal cells and fibroblasts, including PDGF and TGF-β. This would result in a positive feedback loop for myofibroblast formation, excessive extracellular matrix production and eventually fibrosis of the affected tissues. In advanced AMD, formation of a fibrotic disciform scar is associated with resistance to anti-VEGFA therapy and is generally considered to be the final, untreatable end stage of the disease. Our data support the hypothesis that inhibition of EndoMT could suppress the development of this end stage and perhaps expand the therapeutic window of anti-VEGFA therapy for AMD patients in the future.

## Supplementary Materials

The following are available online at https://www.mdpi.com/2073-4409/9/9/2057/s1, Supplementary Table S1: Primers used for qPCR, Supplementary Table S2: List of antibodies used for immunostaining, Figure S1: Optimization of the EndoMT induction protocol, Figure S2: Persistence of EndoMT post-induction, Figure S3: Image intensity and western blot analyses for expression of protein markers by EndoMT and hREC, Figure S4: Activation of p38 MAPK during EndoMT induction, Figure S5: Optimization of SB203580 dosage during EndoMT induction in vitro, Figure S6: The pro-inflammatory microenvironment of the CNV in JR5558 mouse, Figure S7: Expression of vimentin and α-SMA, markers for EndoMT and fibrosis, increase during CNV progression, Figure S8: Raw film images for the western blot analysis.
