# Supplementary material for: Fibrotic Changes and Endothelial-to-Mesenchymal Transition Promoted by VEGFR2 Antagonism Alter the Therapeutic Effects of VEGFA Pathway Blockage in a Mouse Model of Choroidal Neovascularization
Source: Cells. 2020 Sep 9;9(9):2057. doi: 10.3390/cells9092057 (PMC7563259; doi:10.3390/cells9092057)
Supplement: Supplementary file 1 [file cells-09-02057-s001.pdf]

Supplementary information

Supplementary Table 1. Primers used for qPCR

Gene name, species	Forward (5′-3′)	Reverse (5′-3′)
SNAI1, human [25]	CATCCTTCTCACTGCCATGGA	AGGCAGAGGACACAGAACCAGA
SNAI2, human [26]	AGAGCATTTGCAGACAGGTC	AGCCAGATTCCTCATGTTTG
TWIST1, human [27]	GGCTCAGCTACGCCTTCTC	TCCTTCTCTGGAAACAATGACA
ZEB1, human	AGTGATCCAGCCAAATGGAA	TTTTTGGGCGGTGTAGAATC
ZEB2, human	AACAAGCCAATCCCAGGAG	GTTGGCAATACCGTCATCCT
α-SMA, human [27]	CAGGGCTGTTTTCCCATCCAT	ACGTAGCTGTCTTTTTGTCCC
COL3A1, human [28]	AACACGCAAGGCTGTGAGACT	TTTTGTCGGTCACTTGCACTG
VE-cadherin, human [28]	CAGCCCAAAGTGTGTGAGAA	TGTGATGTTGGCCGTGTTAT
VEGFR2, human	CACCACTCAAACGCTGACATGTA	GCTCGTTGGCGCACTCTT
FSP-1, human [28]	GTCCACCTTCCACAAGTAC	TGTCCAAGTTGCTCATCAG
Fibronectin, human [28]	GATAAATCAACAGTGGGAGC	CCCAGATCATGGAGTCTTTA
Vimentin, human [28]	GAGAACTTTGCCGTTGAAGC	TCCAGCAGCTTCCTGTAGGT
COL1A2, human [29]	GCCCCCCAGGCAGAGA	CCAACTCCTTTTCCATCATACTGA
CD31, human [30]	GCGAGTCATGGCCCGAAGGC	GGTGGTGCTGACATCCGCGA
VCAM-1, human [31]	CTGTTGAGATCTCCCCTGGA	CGCTCAGAGGGCTGTCTATC
ICAM-1, human	CAATGTGCTATTCAAACTGCCC	CAGCGTAGGGTAAGGTTCTTG
E-Selectin, human [31]	AGGTTCCTTCCTGCCAAGTGGTAA	ATTGAGCGTCCATCCTTCAGGACA
Tissue factor, human [31]	ACCTCGGACAGCCAACAATTCAGA	ATCCCGGAGGCTTAGGAAAGTGTT
HPRT-1, human [31]	CCTGGCGTCGTGATTAGTGAT	AGACGTTCAGTCCTGTCCATAA
SNAI1, mouse [32]	CTGCTTCGAGCCATAGAACTAAAG	GAGGGGAACTATTGVATAGTCTGT
SNAI2, mouse [32]	CACTCCACTCTCCTTTACC	CAGACTCCTCATGTTTATGC
TWIST1, mouse [32]	CTCGGACAAGCTGAGCAAGATT	ATTTTCTCCTTCTCTGGAAACA
α-SMA, mouse [33]	GGAGAAGCCCAGCCAGTCGC	AGCCGGCCTTACAGAGCCCA
FSP-1, mouse [33]	GGAGCTGCCTAGCTTCCTG	GCTGTCCAAGTTGCTCATCA
Vimentin, mouse [34]	CTGTACGAGGAGGAGATGCG	AATTTCTTCCTGCAAGGATT
Fibronectin, mouse [33]	GAAGTCGCAAGGAAACAAGC	GTTGTAGGTGAACGGGAGGA
COL1A2, mouse [33]	GTCCTAGTCGATGGCTGCTC	CAATGTCCAGAGGTGCAATG
COL3A1, mouse [33]	AGGCCAGTGGCAATGTAAAG	CTCCATTCCCCAGTGTGTTT
IL-6, mouse	CAACCTGAACCTTCCAAAGATG	ACCTCAAACTCCAAAAGACCAG
MCP-1, mouse	TGT CCCAAAGAAGCTGTGATC	ATTCTTGGGTTGTGGAGTGAG
HPRT-1, mouse [19]	TCAGTCAACGGGGGACATAAA	GGGGTCGTACTGCTTAACCAG

Supplementary Table 2. List of antibodies used for immunostaining and western blot

Target / Antibodies Name	Dilution	Source
αSMA	1:200	C6198, Sigma-Aldrich
VE-cadherin	1:200	2500S, Cell Signaling
CD31	1:200	12-0319-41, Invitrogen
phalloidin	1:300	F432, Invitrogen
vimentin	1:200	5741, Cell Signaling
SNAI1	1:200	3879, Cell Signaling
fibronectin	1:200	6328, Abcam
type III collagen	1:300	1330-01, SouthernBiotech
phospho-p38 MAPK	1:200	D3F9, Cell Signaling
NF-kB	1:300	8242, Cell Signaling
IB4-Alexa Fluor™ 488	1:300	I21411, Thermo Fisher Scientific
Rabbit IgG	1:200	39005, Cell Signaling
Goat IgG	1:200	I5256, Sigma
Mouse IgG-Cy3	1:200	Z0109, DAKO
Rat IgG	1:200	I8015, Sigma
Alexa Fluor 647 donkey anti-rabbit IgG	1:500	A31573, Life Technologies
Alexa Fluor 647 donkey anti-goat IgG	1:500	A21447, Life Technologies
Alexa Fluor 594 donkey anti-goat IgG	1:500	A11058, Life Technologies
VEGFR2 (western)	1:1000	9698, Cell Signaling
CD31 (western)	1:1000	3528, Cell Signaling
VE-cadherin (western)	1:1000	2500s, Cell Signaling
phospho-p38 MAPK (western)	1:1000	D3F9, Cell Signaling
p38 MAPK (western)	1:1000	D13E1, Cell Signaling
Alpha-tubulin (western)	1:1000	3873, Cell Signaling
Anti-rabbit IgG, HRP-linked (western)	1:3000	7074s, Cell Signaling
Anti-mouse IgG, HRP-linked (western)	1:3000	7076p2, Cell Signaling

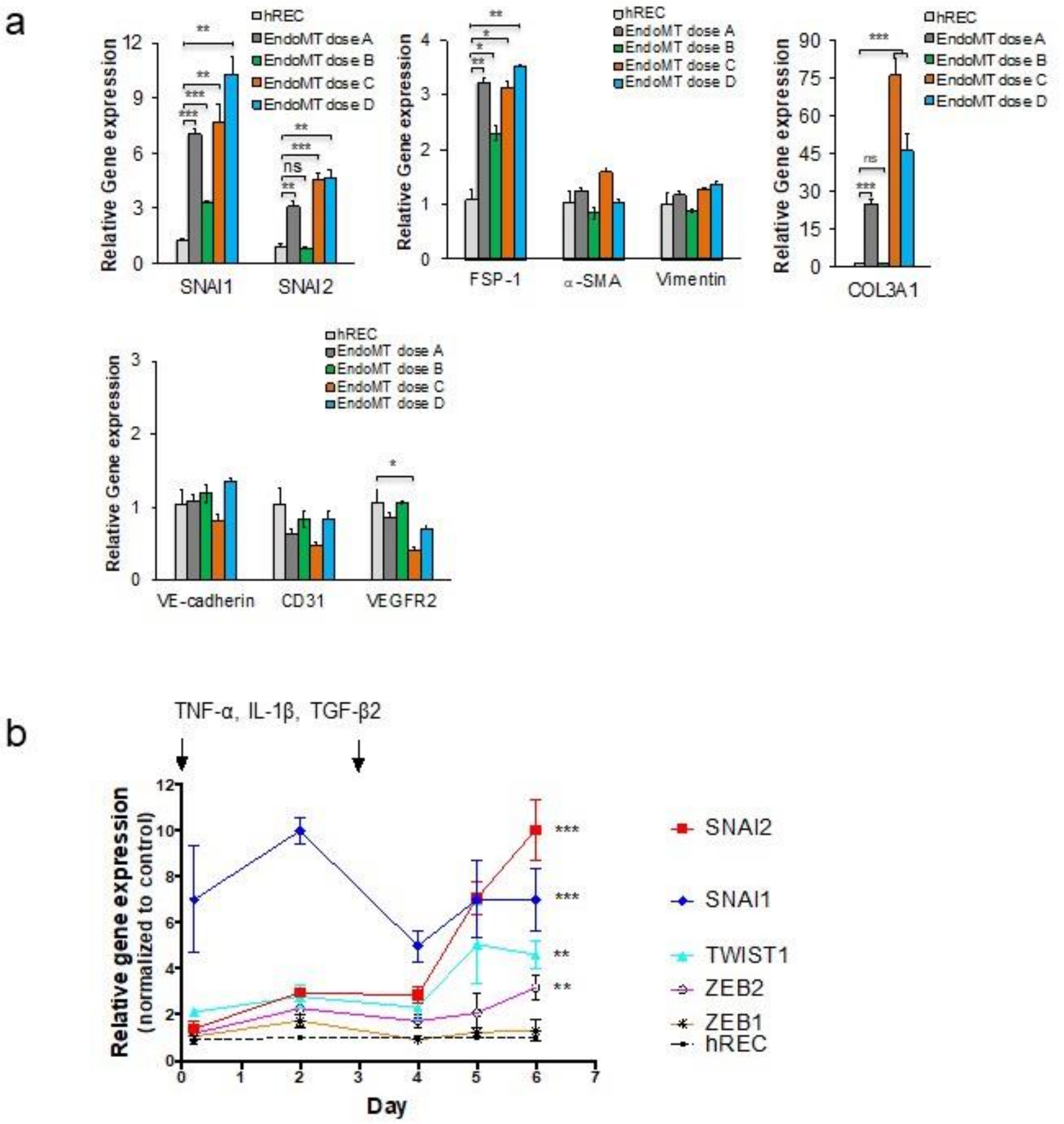

Supplementary Figure S1. Optimization of the EndoMT induction protocol. (a) To optimize the doses of cytokines used for induction of EndoMT, hREC were exposed to different doses of IL-1β, TNF-α and TGF-β2 for six days, then the expression of genes encoding proteins associated with EndoMT (SNAI1, SNAI2, FSP-1, α-SMA, vimentin, COL3A1) and vascular cell differentiation (VE-cadherin, CD31, VEGFR2) was determined by qPCR. Based on these findings, the optimal cytokine doses were determined (see Methods for details). Dose A: 0.05 ng/mL IL-1β, 2.5 ng/mL TNF-α, 2.5 ng/mL TGF-β2; dose B: 0.025 ng/mL IL-1β, 1.25 ng/mL TNF-α, 1.25 ng/mL TGF-β2; dose C: 0.1 ng/mL IL-1β, 2.5 ng/mL TNF-α, 5.0 ng/mL TGF-β2; dose D: 0.1 ng/mL IL-1β, 1.25 ng/mL TNF-α, 5.0 ng/mL TGF-β2. Data = mean ± SEM, *P < 0.05 **P < 0.01, ***P < 0.001, ****P < 0.0001, unpaired 2-tail t-test, comparison between hREC versus EndoMT treated group. *n* = 3. (b) To optimize the time for EndoMT induction, cytokines were applied at the optimal level of 0.1 ng/mL IL-1β, 5.0 ng/mL TNF-α and 5.0 ng/mL TGF-β2 for up to 6 days, and expression levels for genes encoding major transcription factors for EndoMT (SNAI1, SNAI2, TWIST1, ZEB1 and ZEB2) were evaluated by qPCR; hREC not exposed to cytokines were used as a control. Arrows indicate media changes. Data = mean ± SEM, **P < 0.01, ***P < 0.001 compared to the control hREC by unpaired 2-tail t-test, *n* = three independent wells.

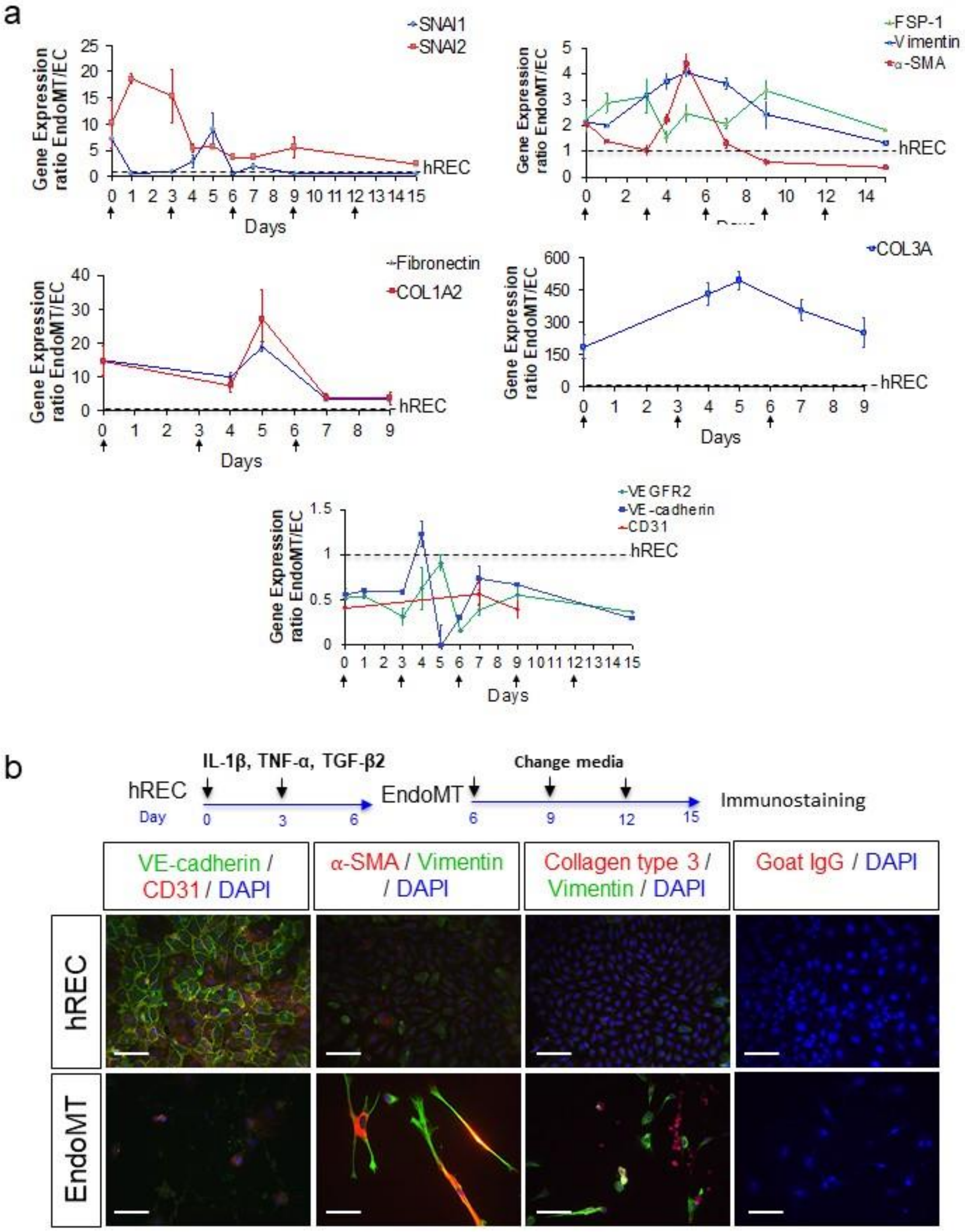

Supplementary Figure S2. Persistence of EndoMT post-induction. (a) Expression of genes encoding proteins associated with EndoMT (SNAI1, SNAI2, FSP-1, vimentin, α-SMA, fibronectin, COL1A2, COL3A) and endothelial cell differentiation (VEGFR2, VE-cadherin, CD31) was assessed over a period of 9-15 days post-induction (T0 = day 6 of EndoMT induction) using qPCR. Arrows indicate media changes and the dotted lines represent the level of expression by the control hREC (normalized to 1). Data = mean ± SEM. n = 3 wells. (b) Localization of proteins associated with EndoMT (vimentin, α-SMA, COL3A) and endothelial cell differentiation (VE-cadherin, CD31) in hREC and EndoMT cells was visualized by immunostaining EndoMT cells at 9 days post-induction and control hREC. Scale bars = 100 μm.

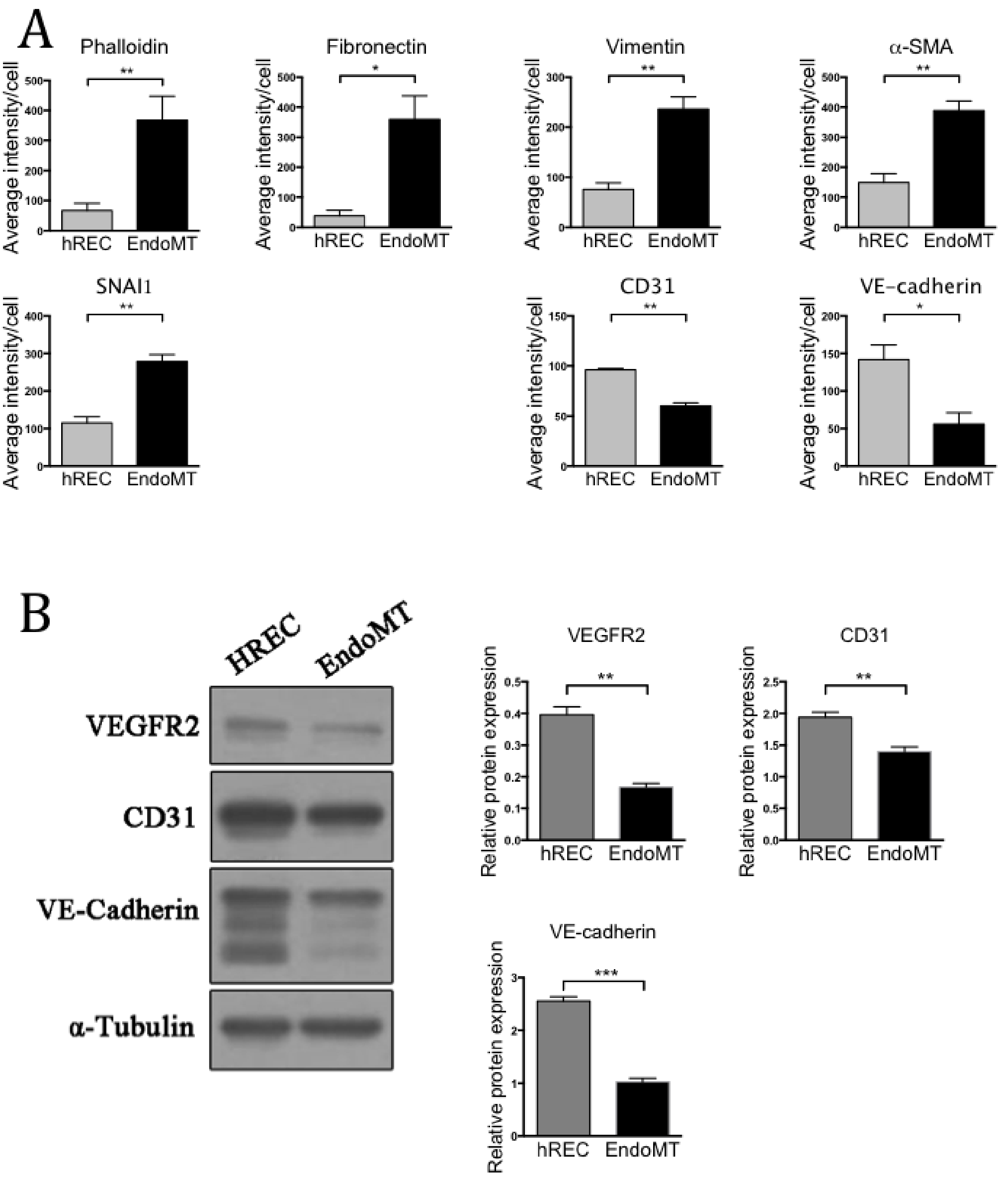

Supplementary Figure S3. Image intensity and western blot analysis for markers of EndoMT and hREC. (A) Cells were fixed and imunostained for various markers of EndoMT and differentiated hREC, and the resulting images were used for intensity quantification using image J. The average intensity of each marker was normalized to the number of nuclei (cells) for each image. Expression levels of EndoMT markers F-actin (phalloidin), fibronectin, vimentin, α-SMA, and SNAI1 were significant increased in the EndoMT cells compared to the hREC, whereas the expression levels of endothelial markers CD31 and VE-cadherin were significantly decreased in the EndoMT cells at 9 days post-induction compared to hREC. (B) Western blot analysis (left, representative micrograph) and densitometry data (right) show significant reductions in protein levels for VEGFR2, CD31 and VE-cadherin in EndoMT cells compared to hREC. Protein levels for each marker were normalized to that of α-tubulin. Data = mean ± SEM, *P < 0.05, **P < 0.01, ***P < 0.001 by unpaired 2-tail t-test, *n* ≥ three independent images/samples from three independent wells per group.

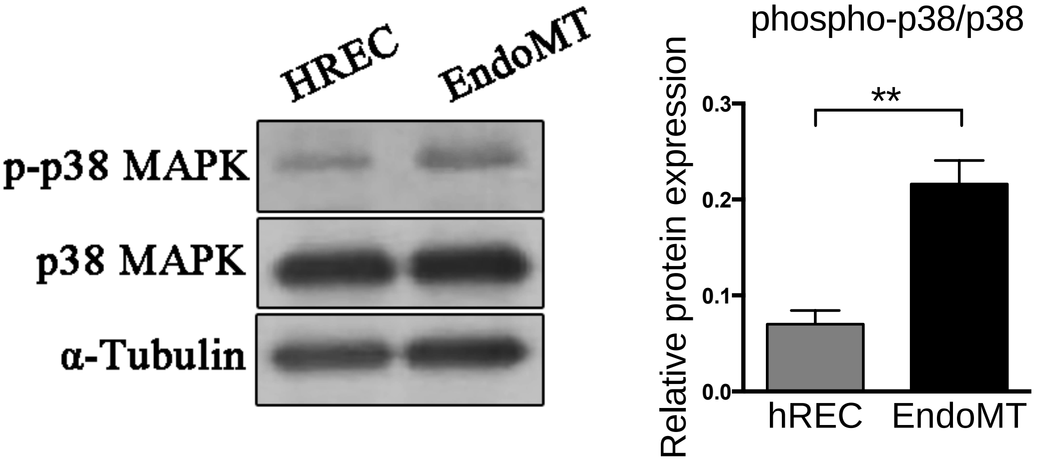

Supplementary Figure S4. Western blot analysis showing significant up-regulation of phospho-p38 MAPK during EndoMT induction compared to hREC. The representative micrograph (left) and densitometry data (right) show a significant increase in the phospho-p38 MAPK/p38 MAPK ratio at 24 hours of EndoMT induction compared to control hREC. Protein levels for each marker were normalized to those of α-tubulin. Data = mean ± SEM, **P < 0.01 by unpaired 2-tail t-test, n = three independent images/samples from three independent wells per group.

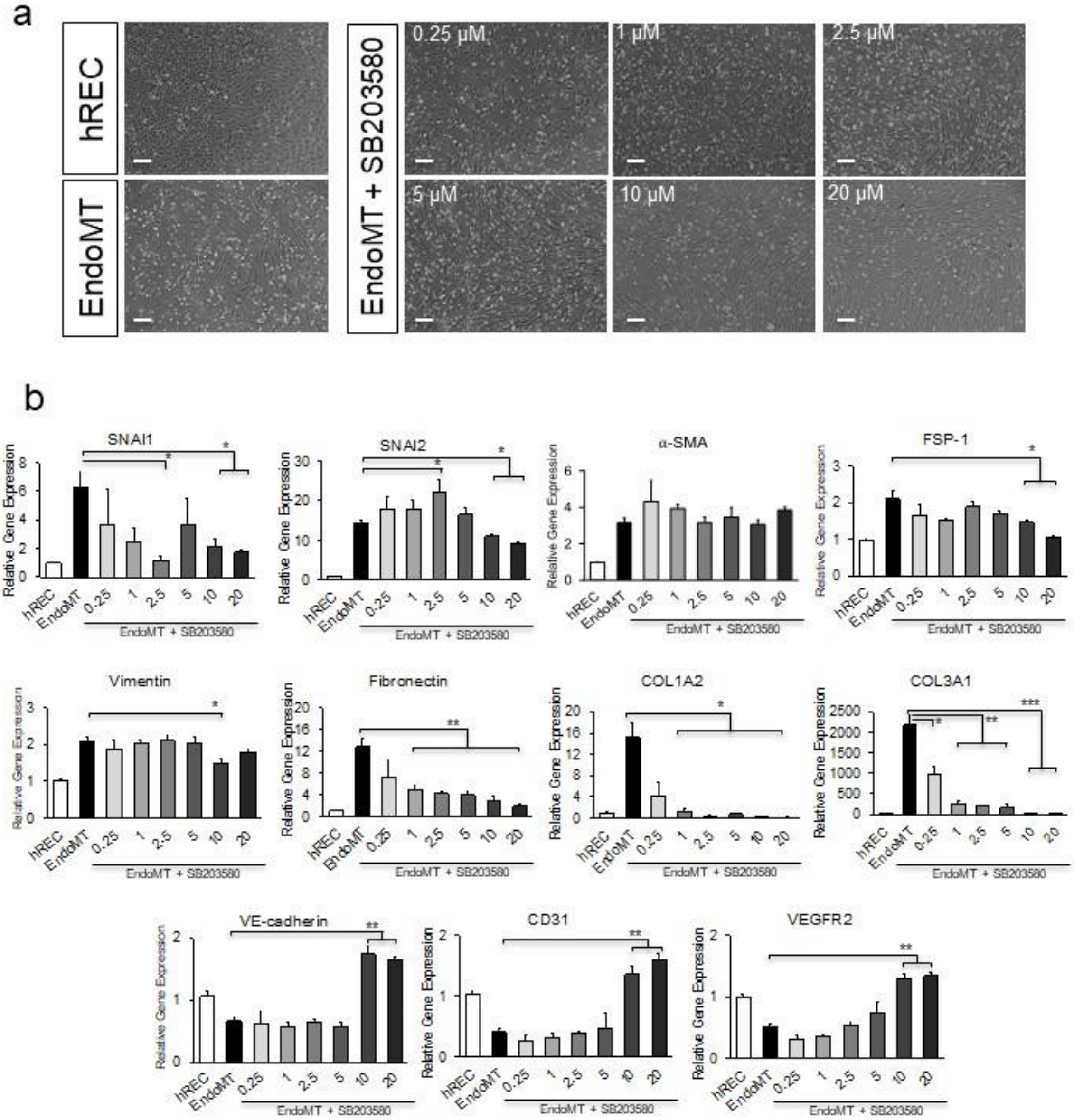

Supplementary Figure S5. Optimization of SB203580 dosage during EndoMT induction in vitro. (a) Phase contrast images show morphology of the control hREC and EndoMT cells with and without doses of SB203580 from 0.5 to 20 μM during EndoMT induction. Scale bars = 100 μm. (b) EndoMT cells were treated during induction with doses of SB203580 ranging from 0.5 to 20 μM, then expression of genes encoding protein markers of EndoMT and endothelial cell differentiation was assessed using qPCR. Data = mean ± SEM, *P < 0.05 **P < 0.01, ***P < 0.001, comparison between hREC versus EndoMT treated group by unpaired 2-tail t-test, *n* = 3 wells.

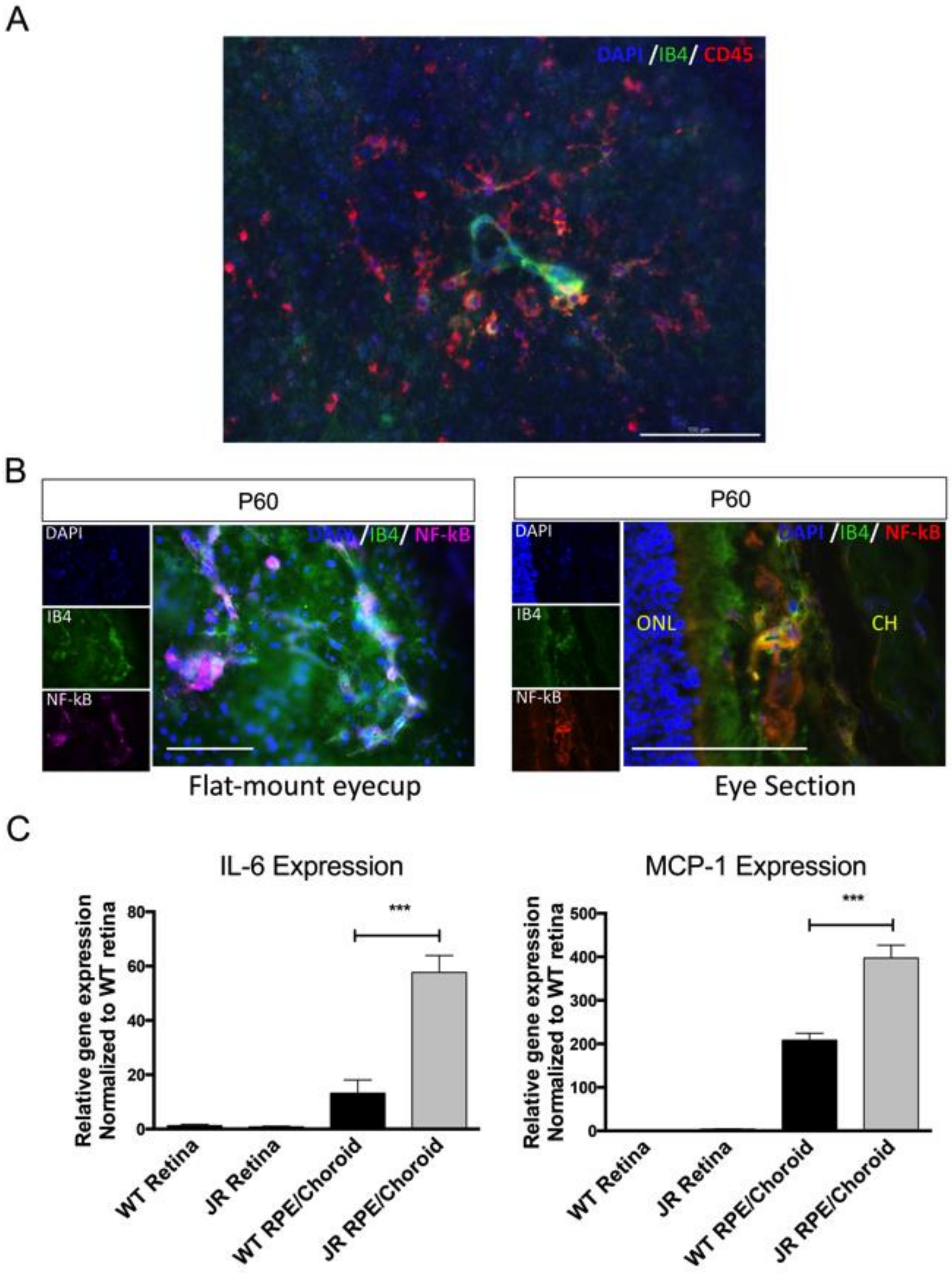

Supplementary Figure S6. The pro-inflammatory microenvironment of the CNV in JR5558 mouse. (a) Flat-mount RPE–choroid eyecup staining showing the recruitment of numerous leukocytes (CD45-positive, red) around the CNV lesion (IB4, green) on top of the RPE layer in the eye of a P31 JR5558 mouse. Scale bar = 100 μm. (b) NF-κB expression in a flat-mount RPE–choroid eyecup (left panels) and RPE–choroid cross section (right panels) in an CNV lesion from a p60 JR5558 mouse was visualized by immunostaining. ONL, outer nuclear layer; CH, choroid layer. Scale bar = 100 μm. (c) Expression of pro-inflammatory genes is higher in the RPE/choroid complex of the JR5558 mouse compared to wild-type (WT) C57BL/6J mouse at P60 based on qPCR analysis. Note that the expression of IL-6 and MCP-1 by the retina samples was comparable between the JR5558 mouse and the WT control. Data = mean ± SEM, ***P < 0.001, one-way ANOVA, *n* = 3 mice (three independent RPE/choroid eyecups).

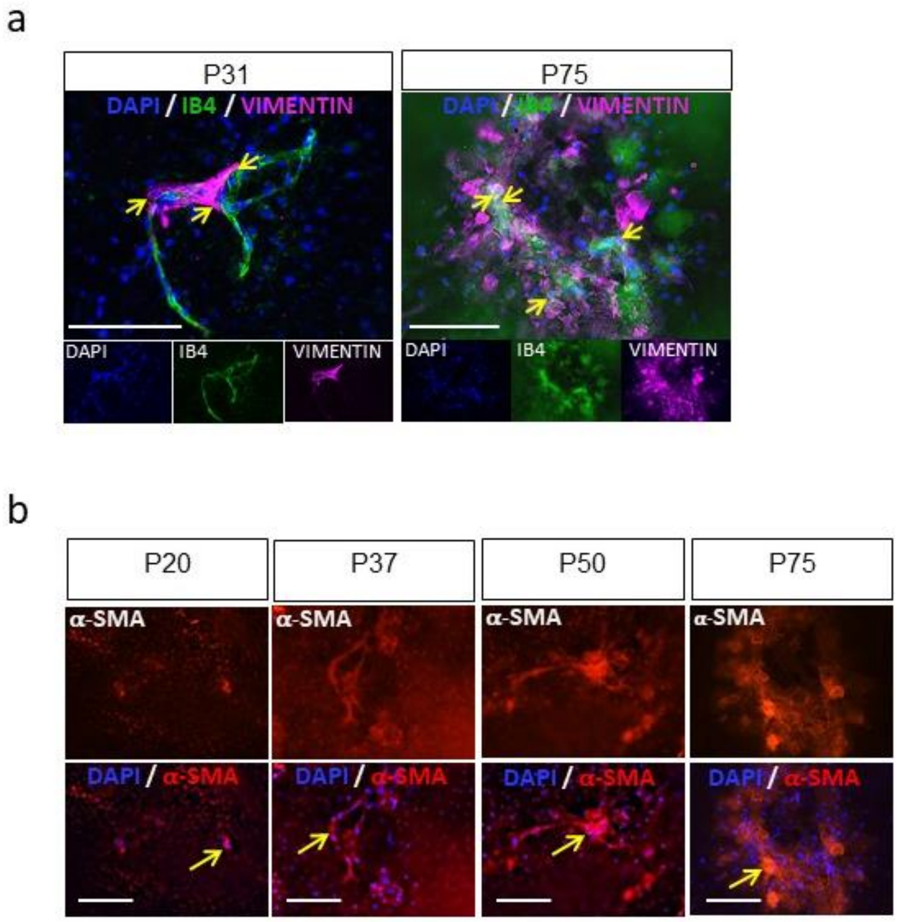

Supplementary Figure S7. Expression of vimentin and α-SMA, markers for EndoMT and fibrosis, increase during CNV progression. (a) Flat-mount RPE–choroid eyecup staining showing the expression of vimentin (purple) by CNV (IB4, green) at P31 and P75. Note that at age P75, many vimentin-positive cells are negative for IB4 (not vessel-associated) as well as positive for IB4 (vessel-associated, arrows), suggesting infiltration of myofibroblasts as the lesion becomes more established. (b) Expression of α-SMA (red) was visualized in vessels of CNV lesions by immunostaining flat mount RPE–choroid eyecups from JR5558 over a time course of CNV progression (P20 to P75). Co-localization of α-SMA with the CNV is shown with the arrow. In micrographs of all panels, scale bars = 100 μm.

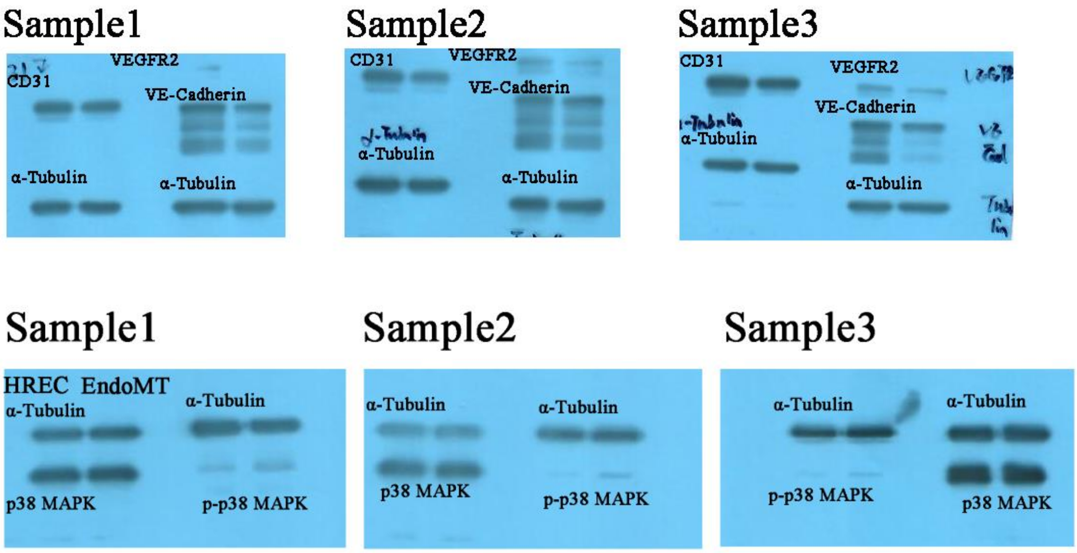

Supplementary Figure S8. Raw film images of western blot analysis. Top row, for Supplementary Figure S3; bottom row, for Supplementary Figure S4
